# Synthesis and structures of gold and copper carbene intermediates in catalytic amination of alkynes

**DOI:** 10.1038/ncomms14625

**Published:** 2017-03-06

**Authors:** Jiwei Wang, Xiaoming Cao, Shichang Lv, Caiyun Zhang, Sheng Xu, Min Shi, Jun Zhang

**Affiliations:** 1 Key Laboratory for Advanced Materials and Institute of Fine Chemicals, School of Chemistry & Molecular Engineering, East China University of Science and Technology, 130 Mei Long Road, Shanghai 200237, China; 2 Center for Computational Chemistry and Research Institute of Industrial Catalysis, School of Chemistry & Molecular Engineering, East China University of Science and Technology, 130 Mei Long Road, Shanghai 200237, China; 3 State Key Laboratory of Organometallic Chemistry, Shanghai Institute of Organic Chemistry, Chinese Academy of Sciences, 354 Fenglin Road, Shanghai 200032, China

## Abstract

Metal carbenes are often proposed as reactive intermediates in the late transition metal-catalysed transformations of alkynes. Owing to their high reactivity, however, isolation and structural characterization of metal carbene intermediates in these transformations has remained unknown. Herein, we report the isolation of two acyclic gold and copper carbene intermediates in either Au(I)- or Cu(I)-catalysed cyclization of *N*-alkynyl formamidines through five-*exo*-dig cyclization. X-ray diffraction, ^13^C NMR spectra data and computational analyses provide evidence for the formation of a gold carbene intermediate with a carbocation-like electronic character. Using the intrinsic bond orbital (IBO) approach, we also evaluate the π-stabilizing effects of organic substituents at the carbene carbon atom in the gold carbene intermediate. Another rare six-membered copper carbene complex is also obtained through 6-*endo*-dig cyclization. These metal carbenes have proven reactive toward oxidation. The metal-promoted cyclization of *N*-alkynyl formamidine provides a facile approach to synthesize metal carbene species.

## Abstract (web summary)

Metal carbenes are typically highly reactive and unstable species, proposed as intermediates in numerous catalytic processes. Here the authors report the isolation and characterization of gold and copper carbene intermediates involved in catalytic cyclization reactions.

Late transition metal carbenes are widely proposed as active intermediate in many catalytic reactions^1^,^2^,^3^,^4^,^5^, and the most common metal carbenes are those prepared by decomposition of an appropriate diazo compound (Fig. 1a)^3^,^4^,^5^. The addition of nucleophilic agents to alkynes catalysed by late transition metals are of profound significance due to their high selectivity and mild conditions and has been investigated extensively for the synthesis of a variety of heterocycles and complex molecules^6^,^7^,^8^. Metal carbenes, in many cases converted from vinyl metal species, are often proposed as key intermediate in these transformations^9^,^10^. In the past 15 years, the transition-metal-catalysed cycloisomerization of ene–yne–ketones has been well established, and provides a safe, effective and practical alternative to the diazo decomposition route for metal carbene generation^11^,^12^,^13^,^14^,^15^,^16^,^17^,^18^,^19^,^20^,^21^,^22^,^23^. In the transformations, the metal π-activated alkyne is attacked by the carbonyl oxygen through 5-*exo*-dig cyclization to generate zwitterionic vinyl metal intermediate **A** that could be transformed into its resonance structure, metal (2-furyl)carbene intermediate **B** (Fig. 1b, M=Cu^19^,^22^, Au^18^,^23^, Rh^13^,^15^, Zn^17^,^18^ and Pd^20^,^21^). Metal carbene **B** subsequently undergoes a variety of transformations, such as oxidation, C-H insertion, cyclopropanation and heteroatom (N, O, Si, S)-H insertion reaction^11^,^12^,^13^,^14^,^15^,^16^,^17^,^18^,^19^,^20^,^21^,^22^,^23^. Recently, the groups of Zhang^18^ and Jiang^19^ reported that both Au(I) and Cu(I) (2-furyl)carbenes (**B** with M=Au(I) or Cu(I)) can undergo carbene oxidation to give 2-acylfurans (Fig. 1b). Like **B**, both Cu(I)-stabilized indazolylcarbene **C** reported by Haley group^24^,^25^,^26^ and Au(I) imidazopyridinylcarbene **D** reported by Cao group^27^ can also trap molecular oxygen to generate the corresponding 3-acylindazole or 3-acylimidazo-pyridine product, respectively. Owing to their high reactivity, however, isolation and structural characterization of late transition metal carbene intermediates in catalytic transformation of alkynes has remained unknown; information regarding the property of the metal carbene intermediates is limited to *in situ* NMR study and computational analysis^28^,^29^. Structurally characterized copper carbenes have been prepared only by either decomposition of the diazo precursors with various copper sources^30^,^31^,^32^,^33^ or carbene transfer from chromium complex^34^. On the other hand, several groups have successfully isolated a variety of structurally characterized gold carbenes^35^,^36^,^37^,^38^,^39^,^40^,^41^,^42^,^43^,^44^,^45^, but there remains an ongoing debate on the extent of the electronic character of gold carbenes as either carbene-like with a strong gold-to-carbon π-backbonding or carbocation-like with a weak π-backbonding^46^,^47^,^48^,^49^,^50^,^51^,^52^,^53^,^54^,^55^. Therefore, structurally characterized copper and gold carbenes, especially those involved in catalytic transformations, are highly desired for a clear understanding of the nature of gold carbenes and their essential role in the catalytic cycle.

As part of our long-standing interest in the design and synthesis of *N*-heterocyclic carbenes (NHCs) and abnormal NHC for catalysis, we previously reported the isolation of a rare vinyl silver species through 6-*endo*-dig cyclization of a formamidine with a σ,π-silver-activated terminal alkyne^56^. Herein, we first report the isolation and characterization of gold and copper carbene intermediates, **E'** (M=Au(I) or Cu(I)), in either Au(I) or Cu(I)-catalysed cyclization oxidation of formamidines with a internal alkyne through 5-*exo*-dig cyclization (Fig. 1c). In the presence of base, Cu(I)-promoted amination of a formamidine with a terminal alkyne leads to form a 6-membered copper carbene through 6-*endo*-dig cyclization. The carbene oxidation reaction of these metal carbene species are directly observed. The detailed structural and spectroscopic studies and computational analysis outline the nature of the gold and copper carbenes.

## Results

### Isolation of active copper carbene intermediates

First, phenyl-substituted formamidine **1** was initially examined in the amination cyclization (Fig. 2a). In the presence of a stoichiometric amount of IPrCuOTf (IPr=2,6-bis(diisopropyl-phenyl)imidazol-2-ylidene), **1** underwent 5-*exo*-dig cyclization to form a 5-membered vinylcopper species **2** at 10 °C in 82% yield, while raising the reaction temperature to 60 °C resulted in 6-*endo*-dig cyclization to afford a 6-membered vinylcopper species **3** in 53% isolated yield. Hydrolysis of either **2** or **3** with HOTf gave the corresponding cyclic formamidinium salt **4** or **5**, respectively. In the presence of a catalytic amount of IPrCuOTf and one equivalent of trifluoromethanesulfonic acid (HOTf), the cyclization of the protonated formamidine **1** was required at elevated temperature to achieve a good yield (90%), forming a 5-membered product **4**. The structures of **4** and **5** were confirmed by X-ray diffraction analysis (see Supplementary Figs 26 and 27).

The Cu-mediated cyclization of formamidine **1** seems to be kinetic versus thermodynamic control reaction. However, attempt to form **3** by heating **2** failed. Vinylcopper species **2** has proven stable only in the solid state at room temperature, and is quite sensitive to oxidation in solution. The formation of a zwitterionic compound **6** was observed from the CDCl_3_ solution of **2** on standing 1 week (Fig. 2b). Compound **6** represents a zwitterionic precursor for a kind of hybrid ambidentate NHC ligand decorating the classical imidazol-2-ylidene with an acetylacetonato unit^57^. Further investigation showed **6** can also be directly obtained via cyclization of **1** using a catalytic amount of IPrCuOTf and H_2_O_2_ as oxidant at 25 °C in 57% isolated yield. When using CuBr.Me_2_S (10 mol%) as catalyst, **6** could be formed in air with a considerable yield (83%). The structure of **6** was confirmed by X-ray crystallography (Fig. 3a), showing it contains a conjugated backbone of alternating single and double bonds of type O1–C2=C3–C16=O2 as evidenced by comparison of bond distances within the acac backbone.

**Figure 1:**
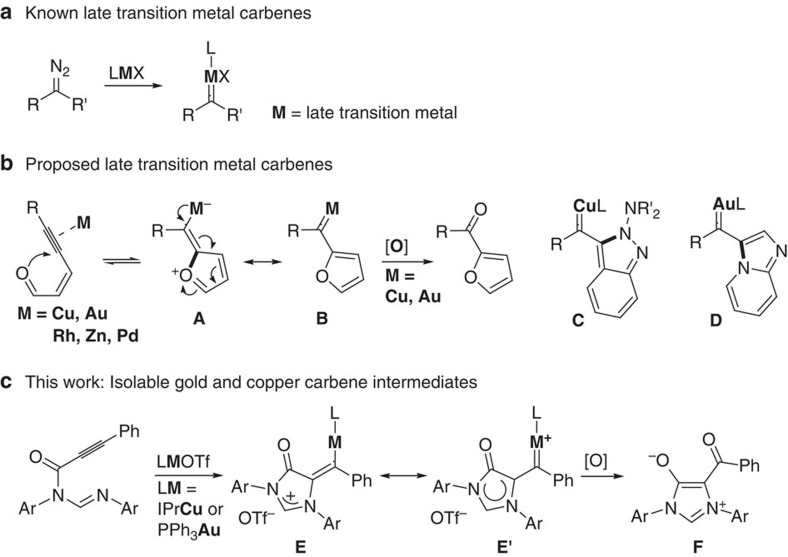
Selected metal carbene species involved in late transition metal-mediated transformations. (**a**) Late transition metal carbenes generated by decomposition of the diazo precursors. (**b**) Proposed late transition metal carbenes in catalytic cycloisomerization of alkynes. (**c**) Gold and copper carbene intermediates isolated in gold- and copper-catalysed cyclization of *N*-alkynyl formamidines.

**Figure 2:**
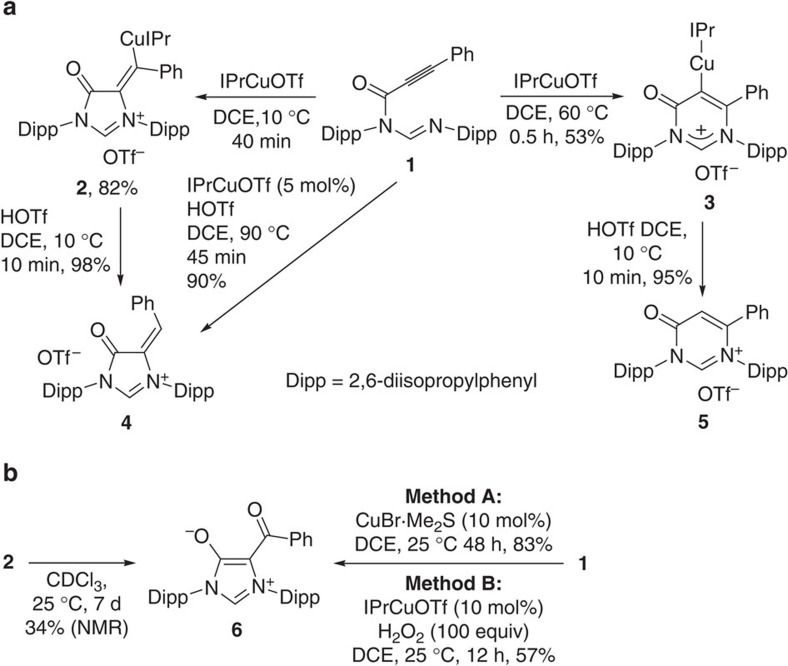
Cu-mediated cyclization of *N*-alkynyl formamidine. (**a**) Cu-promoted amidiniumation of *N*-alkynyl formamidine **1**. (**b**) Cu-catalysed cyclization oxidation of *N*-alkynyl formamidine **1** and transformation of copper intermediate **2** into compound **6**.

The transformation of either vinylcopper species **2** or alkynyl formamidine **1** into **6** is reminiscent of the observation of the carbene oxidation products of both copper (2-furyl)carbene **B** (M=Cu)^19^ and copper pyrazolylcarbene **C**^24^, ^25^,^26^ in the Cu-catalysed cyclization of alkynes (Fig. 1b). Based on the observations, the transformation of **2** into **6** would be better described as a copper carbene oxidation reaction (Fig. 2b). The ^13^C NMR analysis of **2** exhibits one signal for the carbene carbon at *δ*=211.4 p.p.m., which is similar to the imidophosphamidato copper α-carbonyl carbene signal at *δ*=219.0 p.p.m.^32^, upfield of that for the diketiminato copper carbene (*δ*=253.1 p.p.m.)^31^, and fall into the approximate range of 200–400 p.p.m. established for the very deshielded terminal carbene complexes^58^. It is more deshielded than the carbene signal of IPr moiety in **2** at 181.9 p.p.m. The observation verifies the presence of an electrophilic carbene in **2**. Vinylcopper **3** is quite stable in solvent and substantially inert to oxidation. The **3** is colourless, and copper carbene **2** is purple. Similarly, both imidophosphamidato copper α-carbonyl carbenes^30^,^32^ and diketiminato copper carbene complexes^31^ are all violet or purple. It indicates that the presence of a *β*-carbonyl in **2** is crucial for generating a copper carbene intermediate.

**Figure 3:**
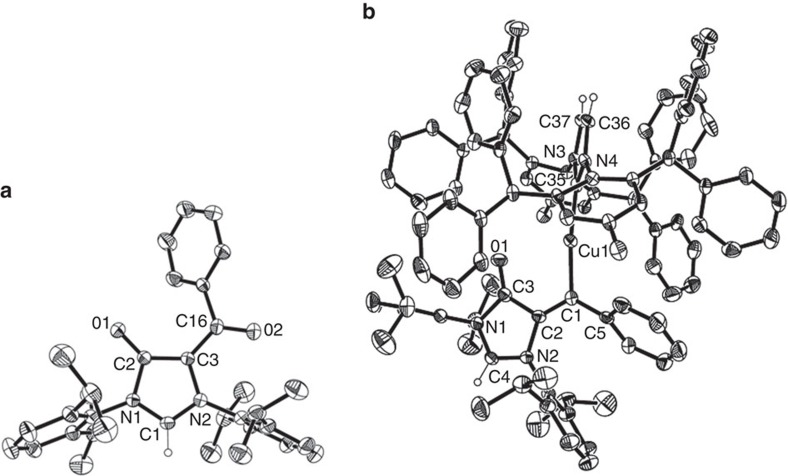
Molecular structure of 6 and 7. (**a**) Molecular structure of **6** with 20% probability. H atoms in aryl rings have been omitted for clarity. (**b**) Molecular structure of **7**·CH_2_Cl_2_ with 20% probability. The counterion (NTf_2_^−^) and CH_2_Cl_2_ in **7** and H atoms in aryl rings have been omitted for clarity.

**Figure 4:**
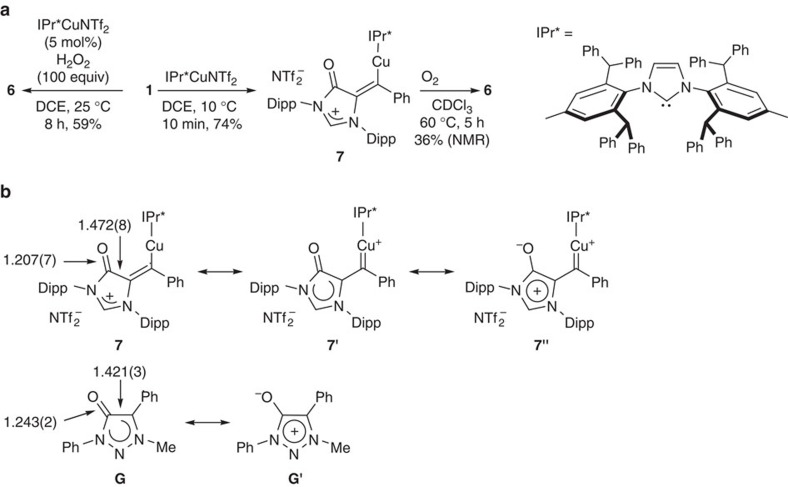
Formation of compound 6 and copper carbene 7 and related mesoionic compound G. (**a**) IPr*CuNTf_2_-catalysed cyclization oxidation of formamidine **1** and related copper carbene **7**. (**b**) Copper carbene complex **7** bearing mesoionic imidazolium-4-olate ring and related mesoionic 1,2,3-triazolium-4-olate **G.**

After massive attempts to isolate single crystals of **2** failed, we managed to obtain a single crystal of its IPr* counterpart copper carbene **7** suitable for X-ray diffraction analysis (Figs 3b and 4a). Copper carbene **7** was prepared as a wine red solid from the cyclization of **1** in the presence of one equivalent of IPr*CuNTf_2_, which could also catalyse the transformation of **1** into **6**. The direct oxidation of **7** to form **6** was also observed in an oxygen atmosphere. In complex **7**, the C-O bond distance of 1.207(7)  Å is characteristic for a C=O double bond. The imidazol-4-one ring in **7** is essentially planar, indicating charge delocalization. The observation suggests the imidazol-4-one ring in **7** could be regarded as a mesoionic imidazolium-4-olate (Fig. 4b). The related mesoionic compounds have been known for several decades, and a similar 1,2,3-triazolium-4-olate compound **G** was reported by Albrecht and co-workers^59^. Therefore, copper complex **7** could be described as a copper carbene complex **7'** bearing a mesoionic imidazolium-4-olate ring. The Cu1-C1 bond (1.924(6) Å) in **7** is similar to that (1.882(3) Å) for a three-coordinate cationic copper carbene [Cu{=CR^1^(OR^2^)}(MeCN)(OEt_2_)]^+^ (R^1^=(*E*)-CH=CH-2-furyl; R^2^=menthyl)^34^, and longer than those (1.822–1.834 Å) observed for the copper carbenes obtained from either diphenyldiazomethane or α-carbonyl diazo compounds^30^,^31^,^32^,^33^. The imidazolium-4-olate ring in **7** is nearly co-planar with the carbene centre, and the neighbouring phenyl ring is tilted with a slightly contracted bond (1.470(8) Å) between the carbene centre and the C_ipso_(Ph) carbon.

### Isolation of active gold carbene intermediate

**Figure 5:**
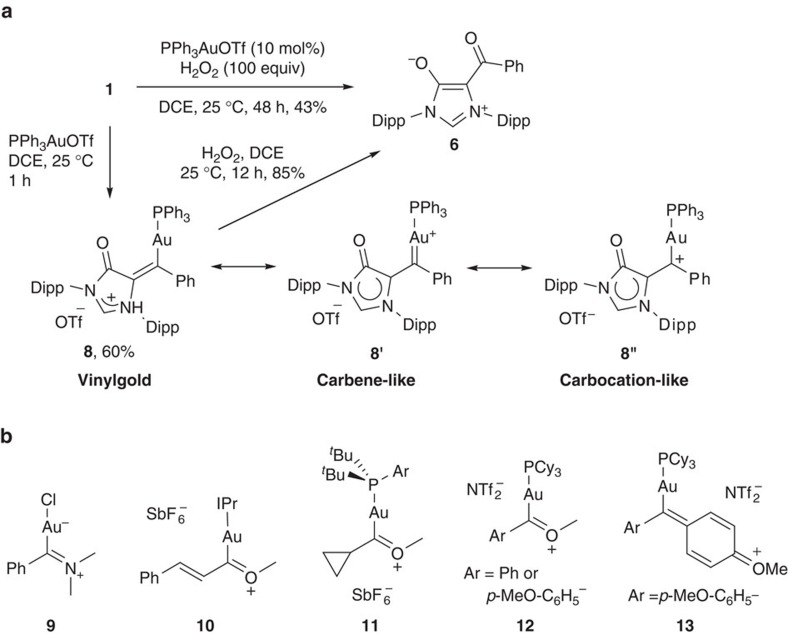
Preparation of gold carbene 8 and related heteroatom-stabilized gold carbenes 9–13. (**a**) Au-catalysed cyclization oxidation of *N*-alkynyl formamidine **1** and gold carbene intermediate **8**. (**b**) Heteroatom-stabilized gold carbenes **9**–**13**.

**Figure 6:**
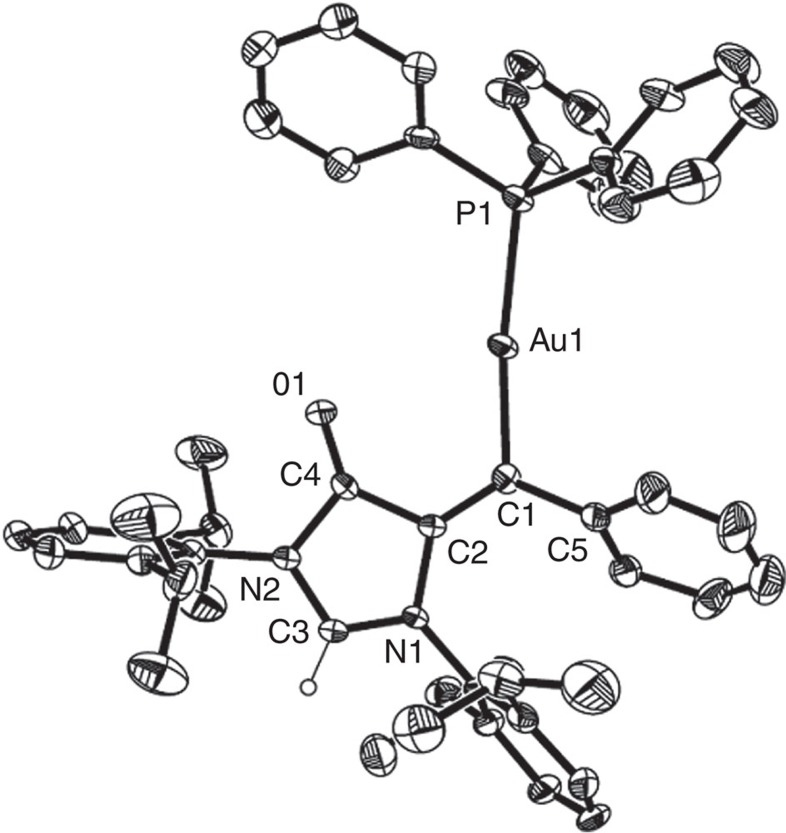
Molecular structure of 8 with 20% probability. The counterion (OTf^−^) and H atoms in aryl rings have been omitted for clarity.

Since gold carbenes are also reactive towards carbene oxidation^16^,^27^, we further investigate gold-catalysed cyclization oxidation of **1**. Delightfully, in the presence of a catalytic amount of PPh_3_AuOTf (10 mol%) and using H_2_O_2_ as oxidant, **6** could be obtained in 43% yield (Fig. 5a). Treatment of a stoichiometric amount of PPh_3_AuOTf with **1** offered gold carbene **8** as a yellow solid in 60% yield. In the presence of H_2_O_2_, **8** underwent oxidation to give **6** in 85% yield. The carbene carbon of **8** in the ^13^C{^1^H} NMR spectra appears as a doublet at *δ*=208.6 p.p.m. (*J*_CP_=103.0 Hz), which is upfield of the carbene carbon resonance of those known gold carbene complexes (*δ*=225∼321 p.p.m.)^35^,^36^,^37^,^38^,^39^,^40^,^41^,^42^,^43^,^44^, and downfield of that for IPrAuCl (*δ*=175.1 p.p.m.)^60^. The formation of **8** was unequivocally confirmed by X-ray crystallography (Fig. 6). Recently, several groups have reported the isolation of gold carbene complexes **9** (ref. 42), **10** (ref. 41), **11** (ref. 37), **12** (refs 35, 36) and **13** (ref. 35), which could be stabilized by π conjugation of the electrodeficient carbene centre with heteroatoms (Fig. 5b). The Au1-C1 bond (2.044(9) Å) in **8** is similar with those found in the gold carbene complexes **12** and **13** (2.039–2.046 Å)^35^,^36^, and longer than those in gold carbene complexes **9**–**11** (2.010–2.032 Å)^37^,^41^,^42^ and non-heteroatom- but diaryl-stabilized gold carbene complexes (1.984(4)–2.014(6) Å)^38^,^40^. Like **7**, the mesoionic imidazolium-4-olate ring in **8** is nearly co-planar with the carbene centre, which enables efficient π-orbital overlap.

### DFT computation and IBO analysis on gold carbene intermediate

**Figure 7:**
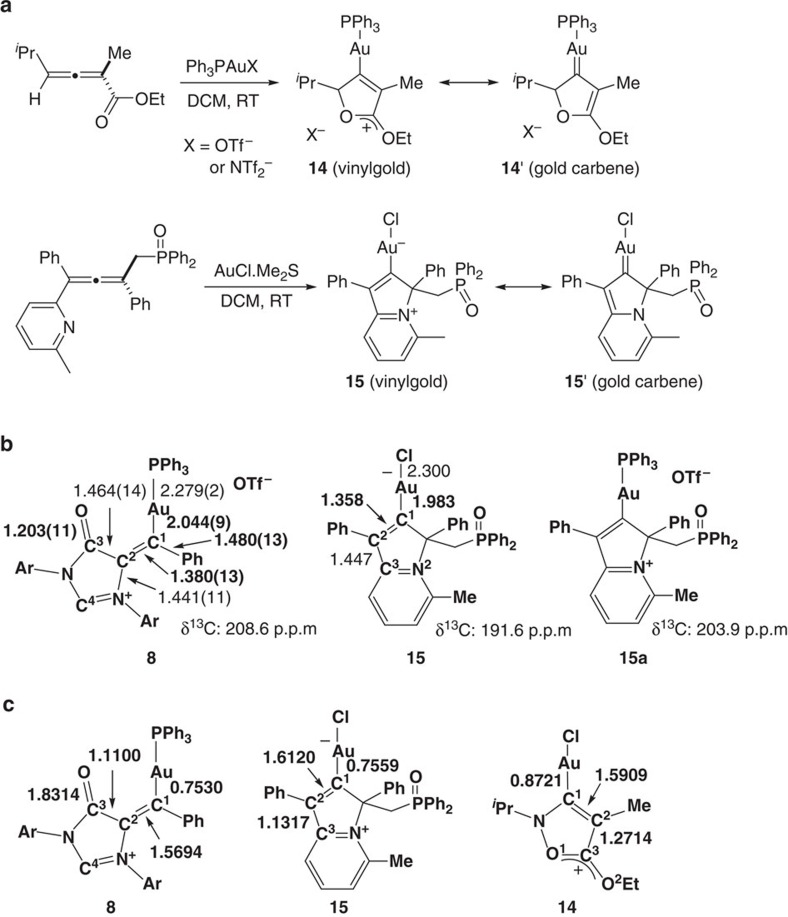
Known gold carbene complexes 14 and 15 and comparison of them with gold carbene 8. (**a**) Previously reported gold carbene complexes **14** (top) and **15** (bottom) prepared through gold-mediated cyclization of allenes. (**b**) Comparison of related bond lengths and characteristic NMR spectroscopic data of complex **8** with those of reference complexes **15** and **15a**; see ref. 63. (**c**) Comparison of Mayer bond order of complex **8** with those of reference complexes **14** and **15**; see refs 63 and 62.

Similar to their copper counterparts, structurally characterized gold carbenes have been mainly prepared by either decomposition of the diazo precursors with various gold sources or carbene transfer from chromium complexes^35^,^36^,^37^,^38^,^39^,^40^,^41^,^42^,^43^,^44^,^45^. Isolation and structural characterization of gold carbene intermediates generated from the addition of nucleophilic agents to C−C multiple bonds remains very rare. In 2008, the Hammond group^61^ reported the spectroscopic detection of a gold carbene/oxonium complex **14** in the gold-mediated cyclization of allenoate and later the Hashmi group^62^ isolated the gold carbene intermediates of type **14** and carried out density functional theory (DFT) calculation to study the bonding properties (Fig. 7). Very recently, Mouries-Mansuy, Fensterbank and colleagues^63^ isolated and structurally characterized a related gold carbene **15** in the gold-mediated cyclization of pyridyl-allene.

The Au–C bond distance of 1.984(2) Å for **15** with a chloride ligand is shorter than that (2.049(9) Å) for **8**, and is in the low range of those known gold carbenes^35^,^36^,^37^,^38^,^39^,^40^,^41^,^42^,^43^,^44^,^45^ (Fig. 7). It should be noted that, in contrast to π-acidic phosphine ligand, the π-donating chloride ligand could increase gold-to-carbon π bonding, resulting in a short Au–C bond^47^. The gold carbene signal of a related PPh_3_-ligated gold carbene **15a** in the ^13^C{^1^H} NMR spectrum appears at *δ*=203.9 p.p.m.^63^, which is similar to that for **8**.

Next, we performed DFT calculations to get more insight into the bonding property in **8**. All the DFT calculations were performed using Gaussian 09 suite of program. The TPSS functional with Grimme's D3-BJ correction for van der Waals interaction was utilized in combination with the triple-ζ basis set def2-TZVPP (see Supplementary Methods for more details). The optimized geometry parameters of **8** are in line with the experimental results. Based on the Mayer bond order analysis (Fig. 7), partial single bond for C^2^−C^1^ can be formulated in the vinylgold complex **8**. Similar bonding scenario was also observed for the related gold complexes **15** (ref. 63) and **14** (ref. 62).

The intrinsic bond orbital (IBO) analysis is a novel method to analyse chemical bonding. The IBOs mainly depict occupied orbitals in an intuitive way, assigning proportionally the electrons in the doubly occupied IBOs to the individual atoms and allowing quantitative interpretation of chemical bonding. Quite recently, using DFT and the IBO approach, Hashmi, Kästner and colleagues^49^,^50^ evaluated the π-stabilizing effects of organic substituents at the carbene carbon atom in several recently isolated and characterized gold carbene complexes, and the observations by the IBO method are consistent with the previously proposed bonding scenario for the gold carbene species. Therefore, we further carried out the IBO approach to study the π-stabilizing effects of organic substituents in gold intermediate **8**.

**Figure 8:**
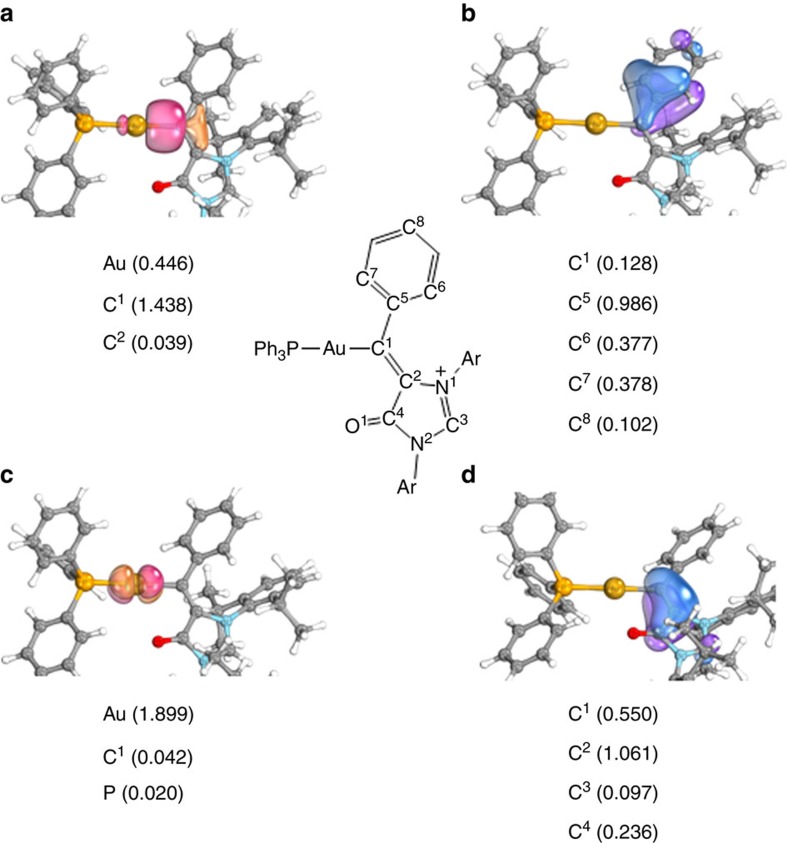
C^1^-stabilizing IBOs of gold complex 8. Numbers in parentheses indicate the fraction of electrons of the doubly occupied orbital assigned to the individual atoms. (**a**) Coordinative bond between the lone pair electrons of C^1^ and Au. (**b**) Delocalized π bond between C^1^ and phenyl ring. (**c**) d-π backbond between Au and C^1^. (**d**) Delocalized π bond between C^1^ and imidazolium-4-olate ring.

As depicted in Fig. 8, we identified a strong π-stabilization in **8**, which is mainly achieved through the π system of the imidazolium-4-olate ring attached to C^1^ (Fig. 8d). Additionally, small contribution from the phenyl ring attached to C^1^ was also identified. The phenyl ring is polarized towards C^1^ (Fig. 8b), forming the delocalized π bonding with C^1^. In addition, the IBO of coordinative bond between the lone pair of carbene C^1^ and the gold atom was identified (Fig. 8a) since this IBO is mainly located at C^1^. The IBO representing the filled d orbital at gold aligned for π backbonding was also identified but it is largely located at gold atom up to 96.8% (Fig. 8c), suggesting little contribution to stabilize carbenic C^1^. Therefore, gold carbene intermediate **8** should be better described as gold- and heteroatom-stabilized carbocation **8″** (Fig. 5).

Very recently, we isolated a related vinylgold complex **16** prepared by reacting IPrAuOTf with a formamidine bearing a terminal alkyne moiety^56^, which could undergo protodeauration to afford a bis(hydroxyimidazol)ium salt (Supplementary Fig. 22). Different from gold carbene **8**, **16** is quite stable in air, while treatment of **16** with excess H_2_O_2_ resulted in complex reaction mixtures without clear product identified. We also reported a PPh_3_ counterpart of **16**, which is prone to undergo aurophilicity to form a vinyl *gem*-digold species^64^. The observations suggest besides the imidazolium-4-olate ring, a phenyl substituent bonding to the carbenic carbon, is also important to stabilize gold carbene **8**. The differences in reactivity between gold species **8** and **16** may possibly imply the gradation in character from carbene to vinylgold species.

### Isolation of six-member copper carbene species

**Figure 9:**
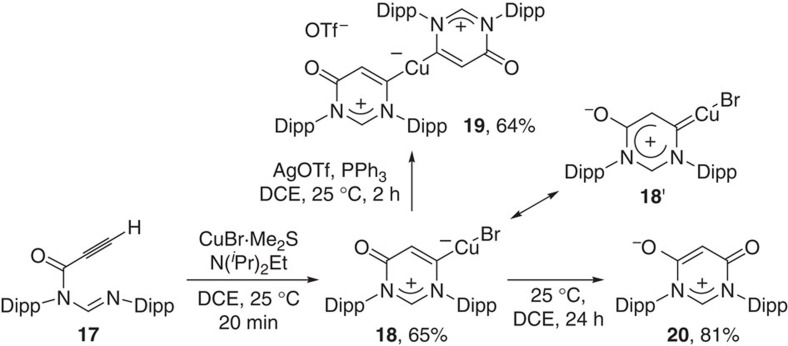
Cu-activated amidiniumation of formamidine 17 having a terminal alkyne in the presence of a base. CuBr-mediated cyclization of formamidine **17** in the presence of N(iPr)_2_Et as a base afforded a 6-membered vinylcopper species **18**, which could be transformed into a divinylcopper species **19**. Vinylcopper **18** is prone to undergo carbene oxidation to give a zwitterionic oxo-adduct **20**, suggesting that **18** is more like a copper carbene species **18**′.

**Figure 10:**
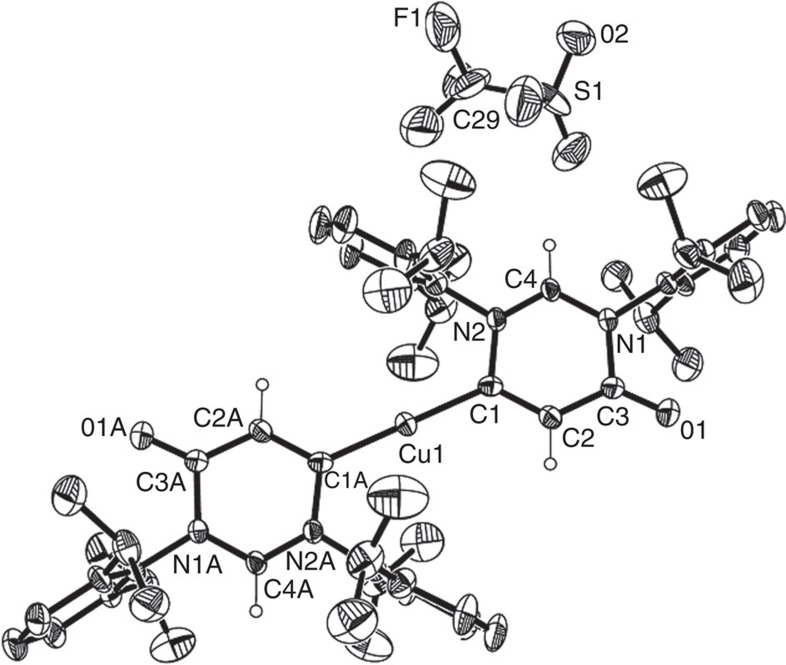
Molecular structure of 19 with 20% probability. H atoms in aryl rings have been omitted for clarity.

In a stoichiometric AgOTf-promoted cyclization of formamidine **17** bearing a terminal alkyne, we previously revealed that presence of a base can preclude the protonation of imino moiety by HOTf, thus changing cyclization fashion^56^. Moreover, Hashmi *et al*.^65^,^66^ also reported that addition of a base can slow down the protodemetallation to conserve organometal species. The observations inspired us to investigate the influence of base in the copper-promoted amidiniumation of **17**. Delightfully, treatment of **17** with CuBr.Me_2_S in the presence of N(^*i*^Pr)_2_Et as a base afforded a 6-membered vinylcopper species **18** as a yellow solid in a 6-*endo*-dig cyclization fashion (Fig. 9). Treatment of **18** with AgOTf in the presence of PPh_3_ resulted in the formation of a divinylcopper species **19** as a yellow solid, the structure of which was confirmed by X-ray crystallography (Fig. 10). The Cu–C bond (1.870(9) Å) in **19** is shorter than that (1.924(6) Å) for copper carbene **7**. In addition, the Cu–C distance in **19** is shorter than those for IPrCuCl (1.953(8) Å)^67^, and the related (IPr_2_Cu)^+^BF_4_^−^ (1.926(19) Å, 1.938(18) Å)^68^, and similar to those for either mesoionic NHC imidazol-5-ylidene copper complex (1.871(7) Å)^69^, or abnormal NHC triazol-5-ylidene copper complex (1.876 (4) Å)^69^. In the ^13^C{^1^H} NMR spectra, the carbene carbon resonates at *δ*=194.5 p.p.m. for **18** and *δ*=193.7 p.p.m. for **19** are similar to the lower range of those established for terminal carbenes (*δ*=200–400 p.p.m.)^58^, and downfield of those for imidazol-5-ylidene copper complex (*δ*=159.5 p.p.m.) and triazol-5-ylidene copper complex (*δ*=166.4 p.p.m.)^69^. Interestingly, similar to the five-membered copper carbene complex **2**, **18** is also found to be reactive towards oxidation in solution. A zwitterionic oxo-adduct **20** was isolated from the DCE solution of **18** on stirring for 1 day, indicating that vinylcopper species **18** is more like a copper carbene complex **18'**, which also contains a *β*-carbonyl. Therefore, we speculate that the reason why the six-membered vinylcopper **3** does not react as a copper carbene is largely because of the presence of an *α*-carbonyl instead of a *β*-carbonyl in **2** and **18**. Zwitterionic compound **20** is known as a zwitterionic precursor for an amino-acrylamido carbene^70^.

## Discussion

We have developed both Au(I)- and Cu(I)-catalysed cyclization oxidation of *N*-propiolic formamidines through 5-*exo*-dig cyclization. Key gold and copper carbene intermediates are isolated from the stoichiometric reaction, which were fully characterized. X-ray diffraction analysis and ^13^C NMR spectra data provide evidence for the formation of a gold carbene intermediate with a carbocation-like electronic character. Using DFT and the IBO approach, we evaluated the π-stabilizing effects of organic substituents at the carbene carbon atom in the gold carbene complex **8**. In the presence of base, Cu(I)-promoted amidiniumation of the formamidine with a terminal alkyne leads to form a six-membered copper carbene through 6-*endo*-dig cyclization. The generation of these gold and copper carbene complexes is attributed to the presence of a *β*-carbonyl group, and their oxidation reactions are directly observed. The metal-promoted cyclization reaction of *N*-alkynyl formamidines also provides a facile approach to synthesize metal carbene species, and our future efforts are directed at synthesizing other metal species by using the facile method.

## Methods

### General

Unless otherwise stated, all reactions and manipulations were performed using standard Schlenk techniques. All solvents were purified by distillation using standard methods. Commercially available reagents were used without further purification. NMR spectra were recorded by using a Bruker 400 MHz spectrometer. Chemical shifts are reported in p.p.m. from tetramethylsilane with the solvent resonance as the internal standard (^1^H NMR CDCl_3_: 7.26 p.p.m.; ^13^C NMR CDCl_3_: 77.0 p.p.m.; ^13^C NMR DMSO: 39.43 p.p.m.). Synthetic procedures for compounds **1c**, **2**–**6** and **18**–**20** are summarized in Supplementary Methods. For NMR analysis and X-ray structures of the compounds in this article, see Supplementary Figs 1–21. For more details, please see also Supplementary Methods.

### Synthesis of copper carbene complex 7

The mixture of formamidine **1** (100 mg, 0.20 mmol) and IPr*CuNTf_2_ (205 mg, 0.20 mmol) was stirred in the 1,2-dichloroethane (DCE; 3 ml) at 10 °C for 30 min. All volatiles were removed under vacuum, and the rude product was washed twice with diethyl ether to afford pure **7** as a wine red solid (260 mg, 74%). ^1^H NMR (400 MHz, CDCl_3_) *δ*=9.07 (s, 1H), 7.64 (t, *J*=7.9 Hz, 1H), 7.40 (d, *J*=7.9 Hz, 2H), 7.17-7.07 (m, 19H), 7.02-6.97 (m, 3H), 6.90-6.84 (m, 14H), 6.76 (d, *J*=7.6 Hz, 7H), 6.71 (s, 4H), 6.45 (t, *J*=6.8 Hz, 1H), 6.27 (t, *J*=7.6 Hz, 2H), 5.80 (d, *J*=7.6 Hz, 2H), 5.48 (s, 2H), 5.20 (s, 4H), 2.83-2.76 (m, 2H), 2.74-2.65 (m, 2H), 2.15 (s, 6H), 1.33 (d, *J*=6.8 Hz, 6H), 1.27-1.18 (m, 12H), 1.13 (d, *J*=6.8 Hz, 3H), 1.05 (d, *J*=6.8 Hz, 3H); ^13^C NMR (100 MHz, CDCl_3_) *δ*=215.41, 181.18, 163.28, 151.88, 145.91, 144.13, 143.18, 142.38, 140.81, 140.19, 139.46, 134.48, 131.66, 129.99, 129.55, 129.43, 129.38, 129.18, 128.68, 128.43, 128.06, 126.79, 126.55, 126.24, 124.80, 124.57, 124.41, 124.24, 123.69, 29.79, 29.24, 26.00, 24.60, 23.66, 22.42, 21.99, 21.36; HRMS (MALDI): m/z [M-NTf_2_]^+^ calcd. for C_103_H_96_CuN_4_O^+^: 1467.6880; found: 1467.6895.

### Synthesis of gold carbene complex 8

The mixture of PPh_3_AuCl (99 mg, 0.20 mmol) and silver triflate (51 mg, 0.20 mmol) was stirred in the DCE (1.5 ml) at 25 °C for 15 min, and then the solid components were filtered off and the filtrate was added to the solution of **1** (100 mg, 0.20 mmol) in the DCE (1 ml). After stirring for 1 h at 25 °C, all volatiles were removed under vacuum. The rude product was washed twice with diethyl ether to afford pure **8** as a yellow solid (132 mg, 60%). ^1^H NMR (400 MHz, CDCl_3_) *δ*=10.19 (s, 1H), 7.56-7.47 (m, 10H), 7.46-7.39 (m, 6H), 7.35 (d, *J*=7.9 Hz, 3H), 6.96 (d, *J*=7.9 Hz, 2H), 6.93-6.83 (m, 5H), 2.84-2.67 (m, 4H), 1.39-1.26 (m, 18H), 1.22 (d, *J*=6.8 Hz, 6H); ^13^C NMR (100 MHz, CDCl_3_) *δ*=208.56 (d, ^2^*J*_C-P_=103.0 Hz), 162.98, 155.86, 145.75, 144.33, 140.93, 134.16, 131.66, 131.17, 129.60, 129.16, 127.12, 125.70, 125.27, 124.61, 124.21, 121.89, 29.62, 29.22, 25.96, 24.36, 23.62, 21.84; ^31^P NMR (162 MHz, CDCl_3_) *δ*=39.52; HRMS (MALDI): m/z [M-OTf]^+^ calcd. for C_52_H_55_AuN_2_OP^+^: 951.3718; found: 951.3699.

### Data availability

The X-ray crystallographic coordinates for structures reported in this study have been deposited at the Cambridge Crystallographic Data Centre (CCDC), under deposition numbers CCDC 1418063 (**4**), CCDC 1418064 (**5**), CCDC 1449714 (**6**), CCDC 1470533 (**7**), CCDC 1470532 (**8**) and CCDC 1449046 (**19**). These data can be obtained free of charge from the Cambridge Crystallographic Data Centre via www.ccdc.cam.ac.uk/data_request/cif. All data are available from the authors on reasonable request.

## Additional information

**How to cite this article:** Wang, J. *et al*. Synthesis and structures of gold and copper carbene intermediates in catalytic amination of alkynes. *Nat. Commun.* **8,** 14625 doi: 10.1038/ncomms14625 (2017).

**Publisher's note:** Springer Nature remains neutral with regard to jurisdictional claims in published maps and institutional affiliations.

## Supplementary Material

Supplementary Information Supplementary figures, supplementary tables, supplementary methods and supplementary references.
