# Supplementary material for: Synthesis and structures of gold and copper carbene intermediates in catalytic amination of alkynes
Source: Nat Commun. 2017 Mar 6;8:14625. doi: 10.1038/ncomms14625 (PMC5343500; doi:10.1038/ncomms14625)
Supplement: Supplementary Information — Supplementary figures, supplementary tables, supplementary methods and supplementary references. [file ncomms14625-s1.pdf]

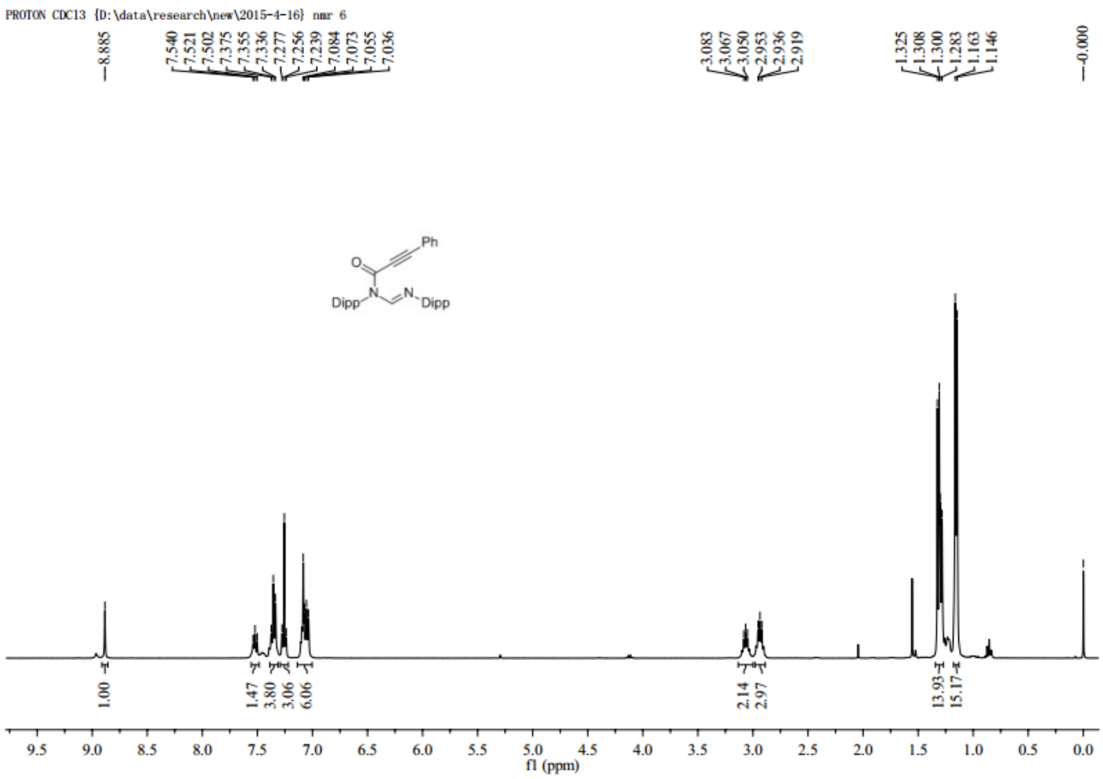

Supplementary Figure 1: ^{1}H-NMR Spectra of compound 1c

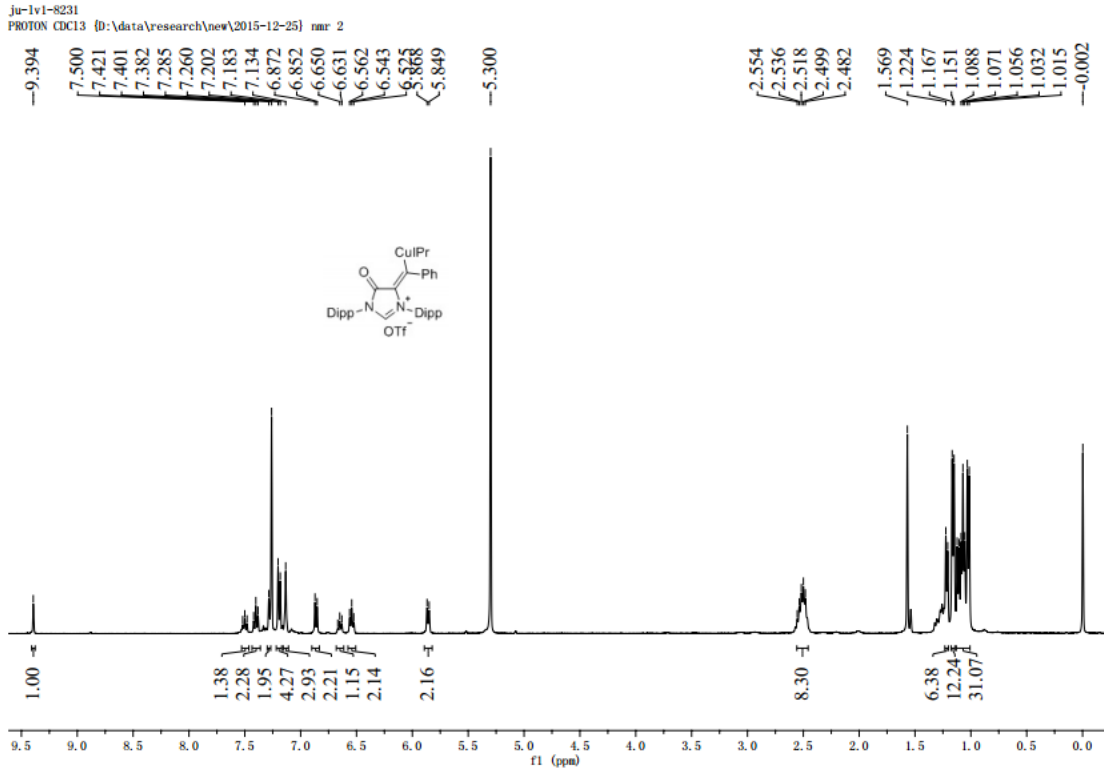

Supplementary Figure 2: ^{1}H-NMR Spectra of complex 2

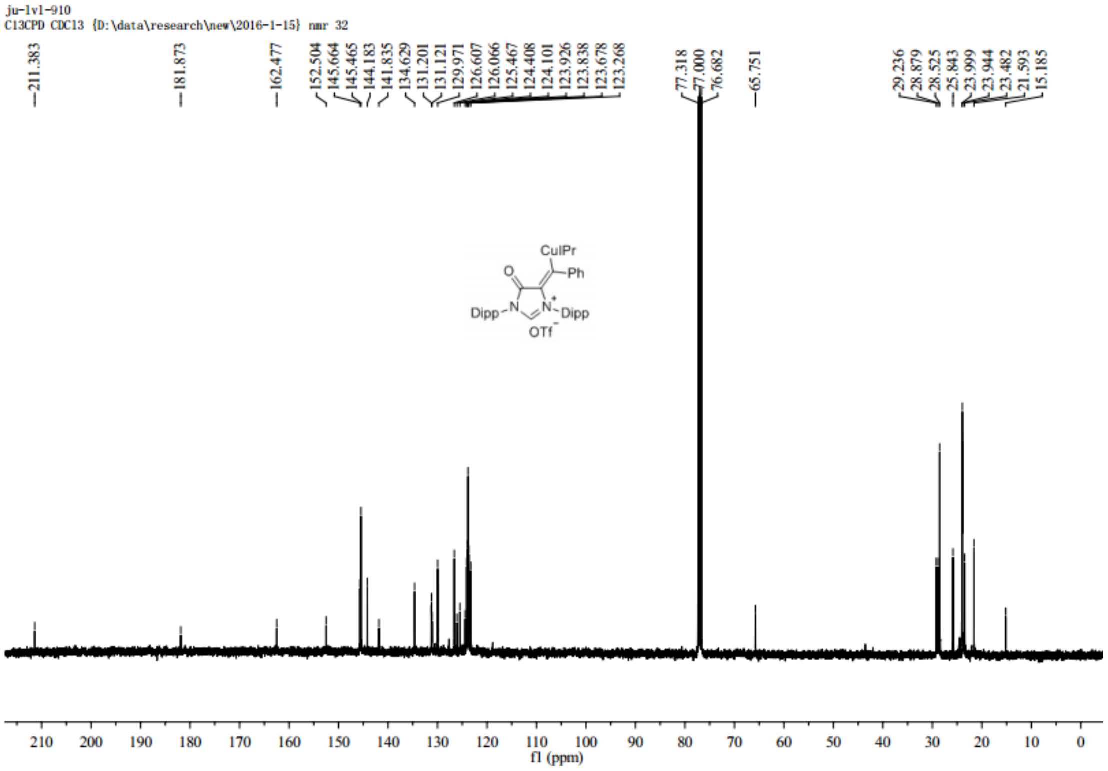

Supplementary Figure 3: ^{13}C-NMR Spectra of complex 2

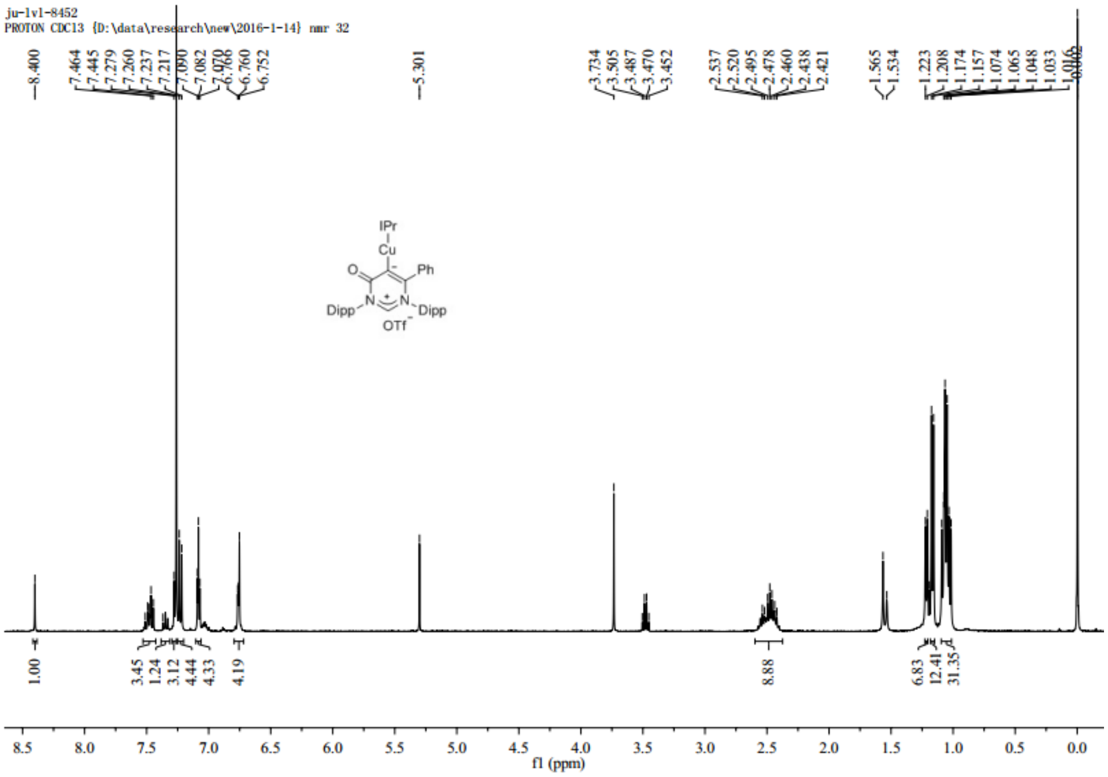

Supplementary Figure 4: ^{1}H-NMR Spectra of complex 3

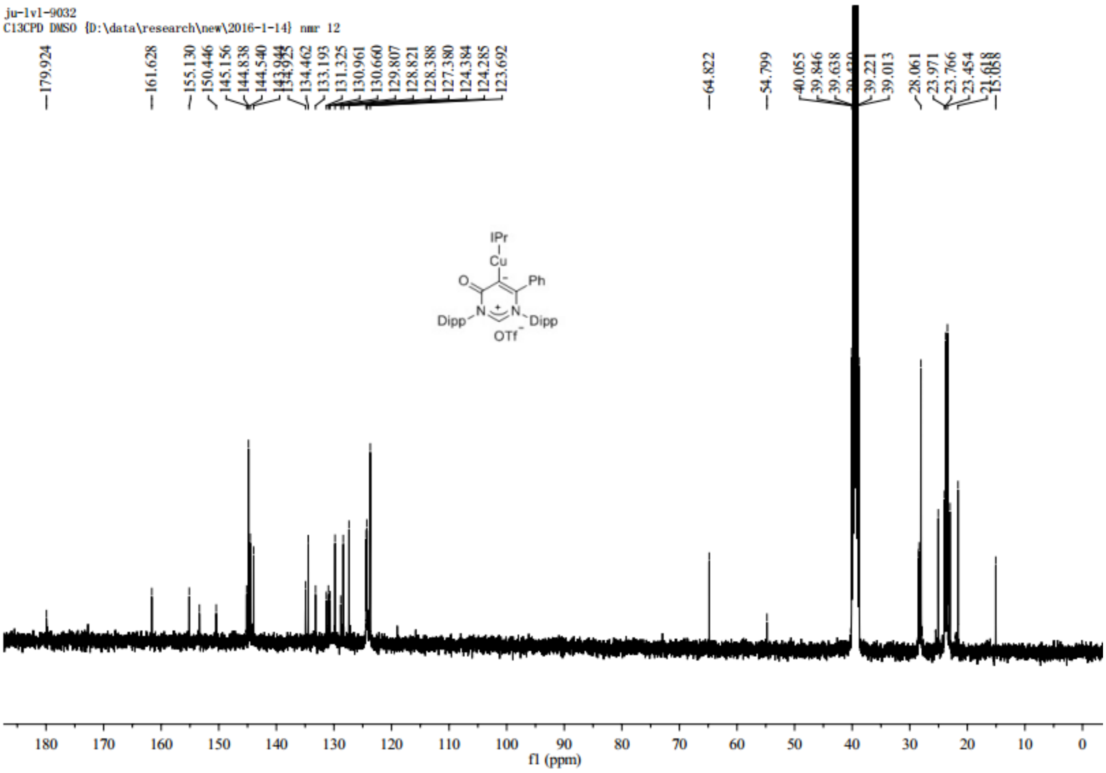

Supplementary Figure 5: ^{13}C-NMR Spectra of complex 3

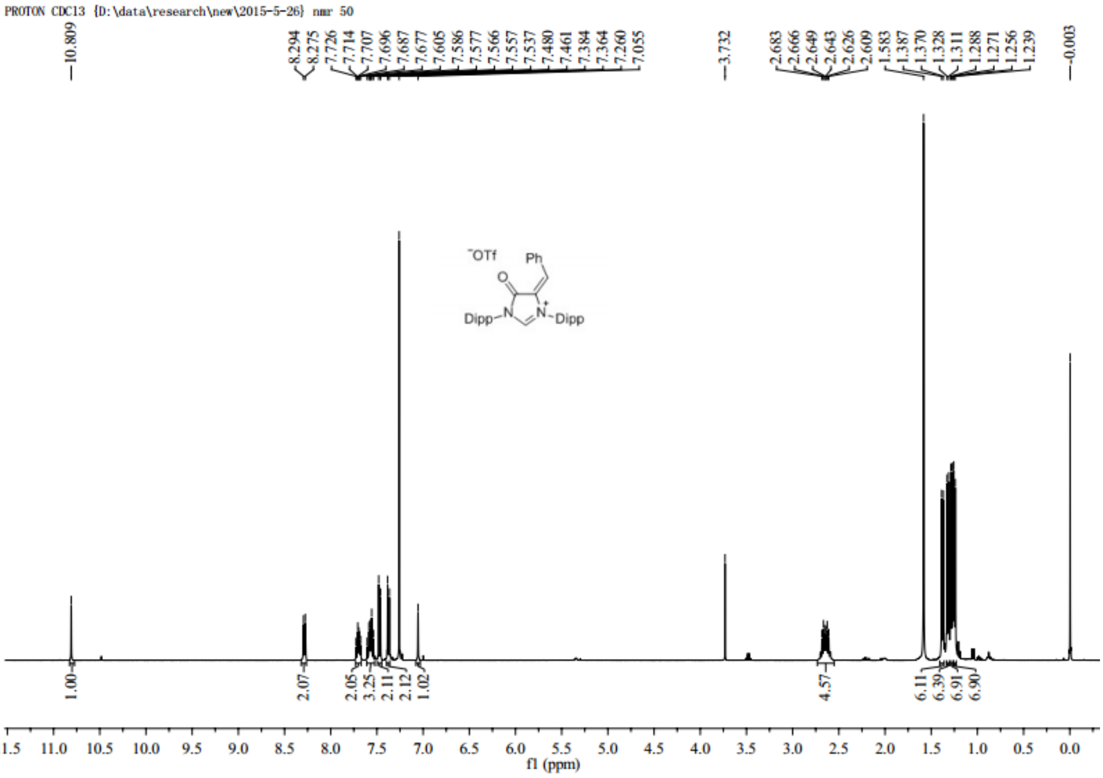

Supplementary Figure 6: ^{1}H-NMR Spectra of compound 4

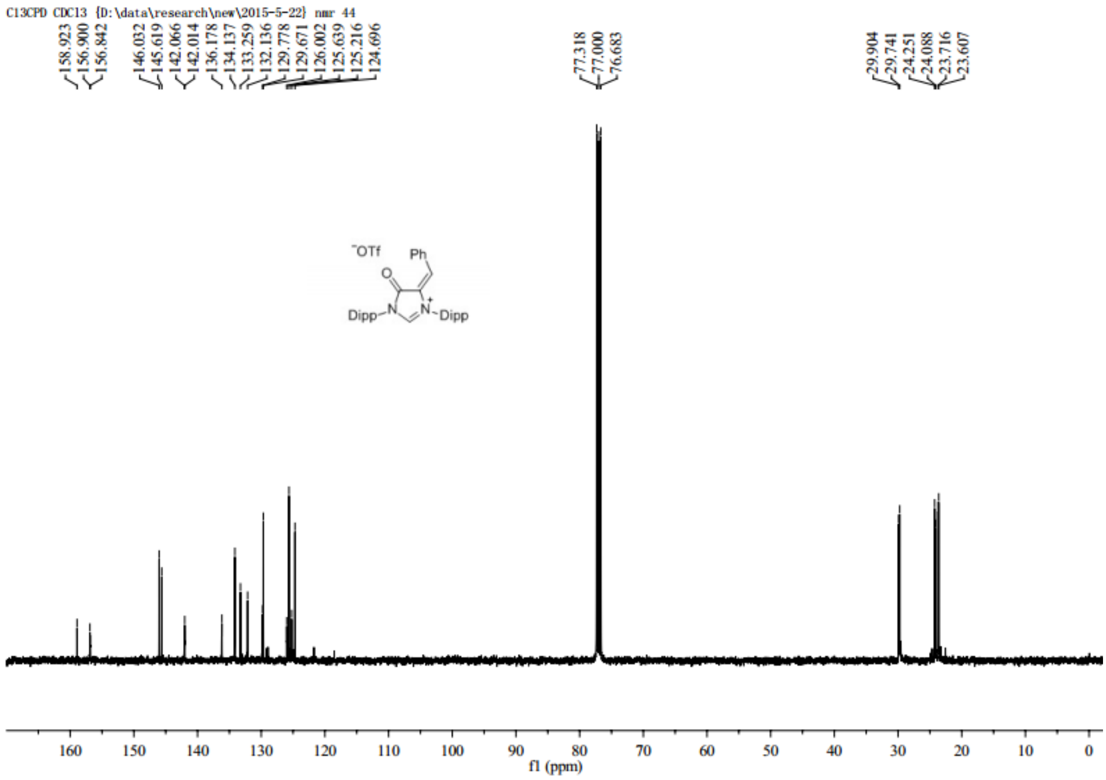

Supplementary Figure 7: ^{13}C-NMR Spectra of compound 4

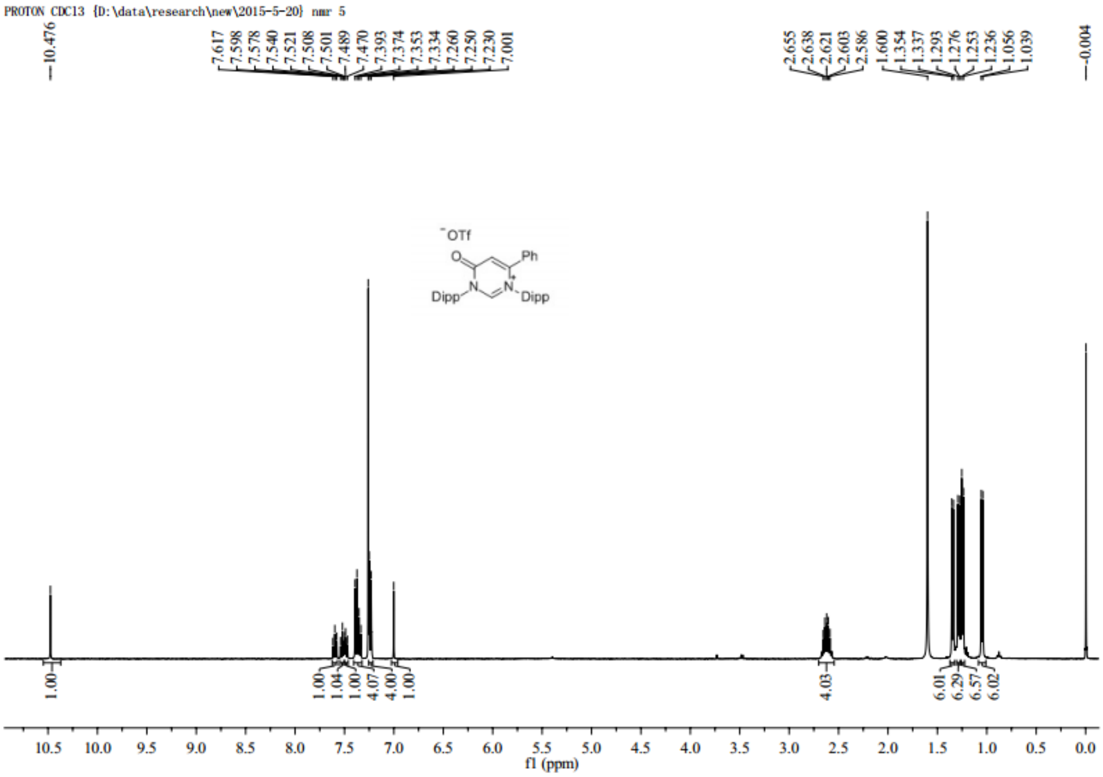

Supplementary Figure 8: ^{1}H-NMR Spectra of compound 5

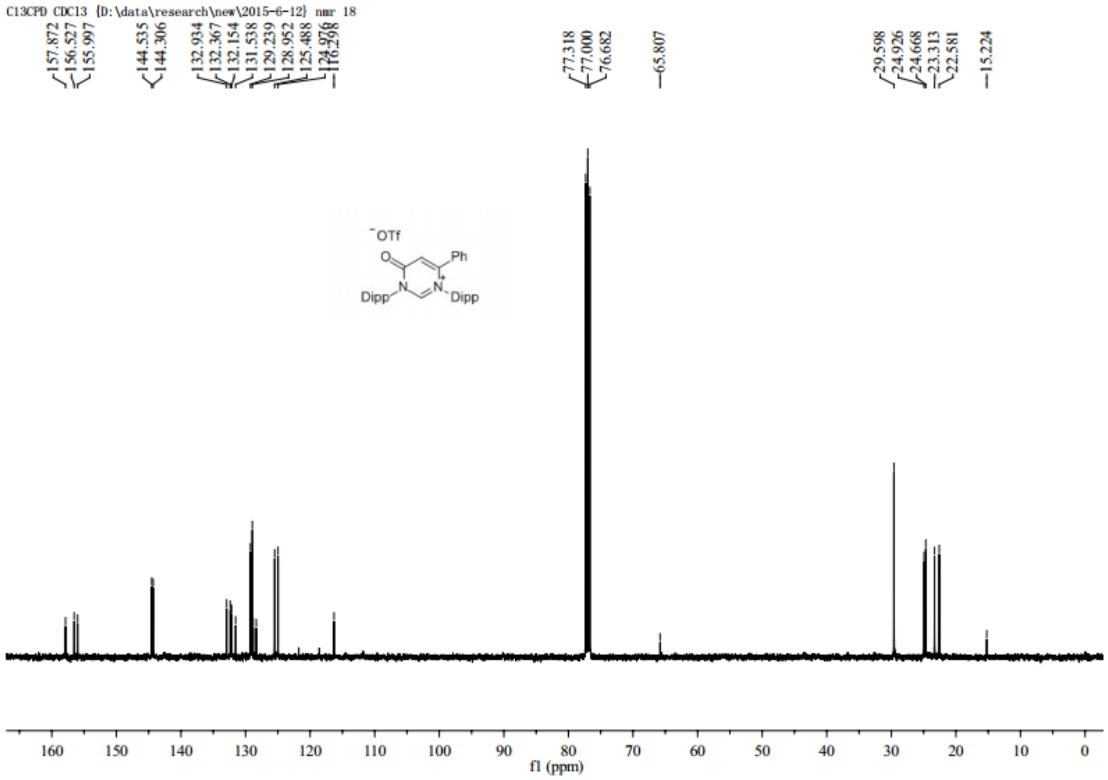

Supplementary Figure 9: ^{13}C-NMR Spectra of compound 5

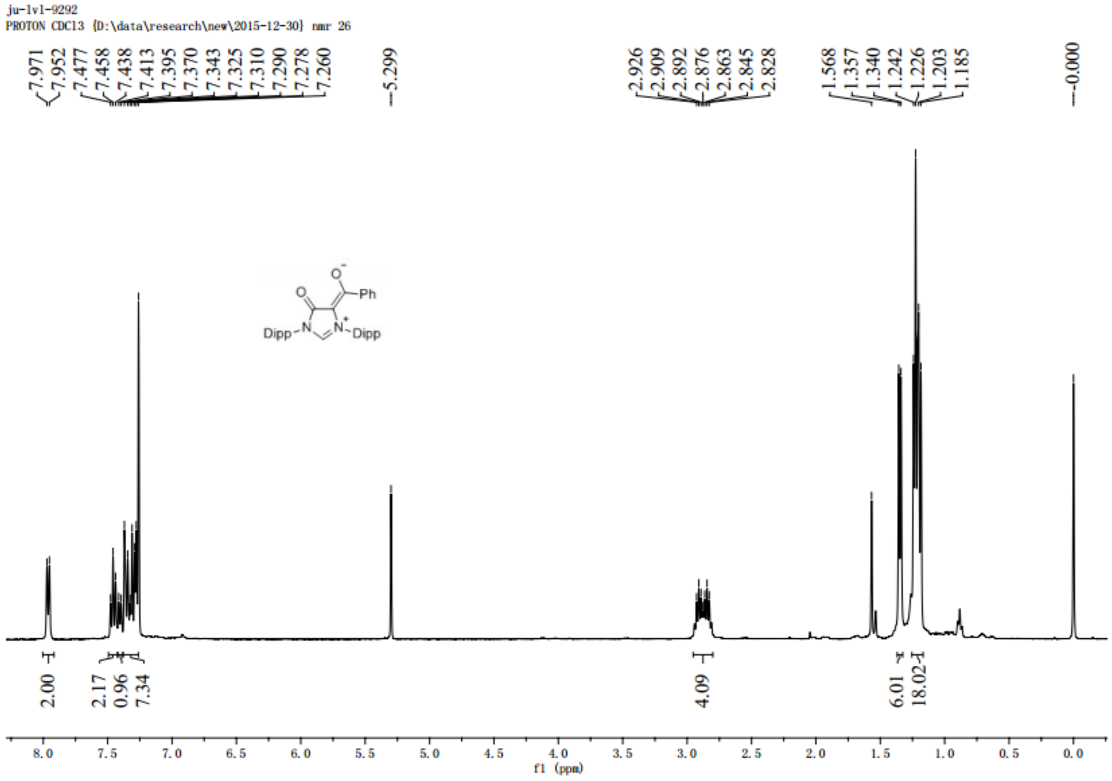

Supplementary Figure 10: ^{1}H-NMR Spectra of compound 6

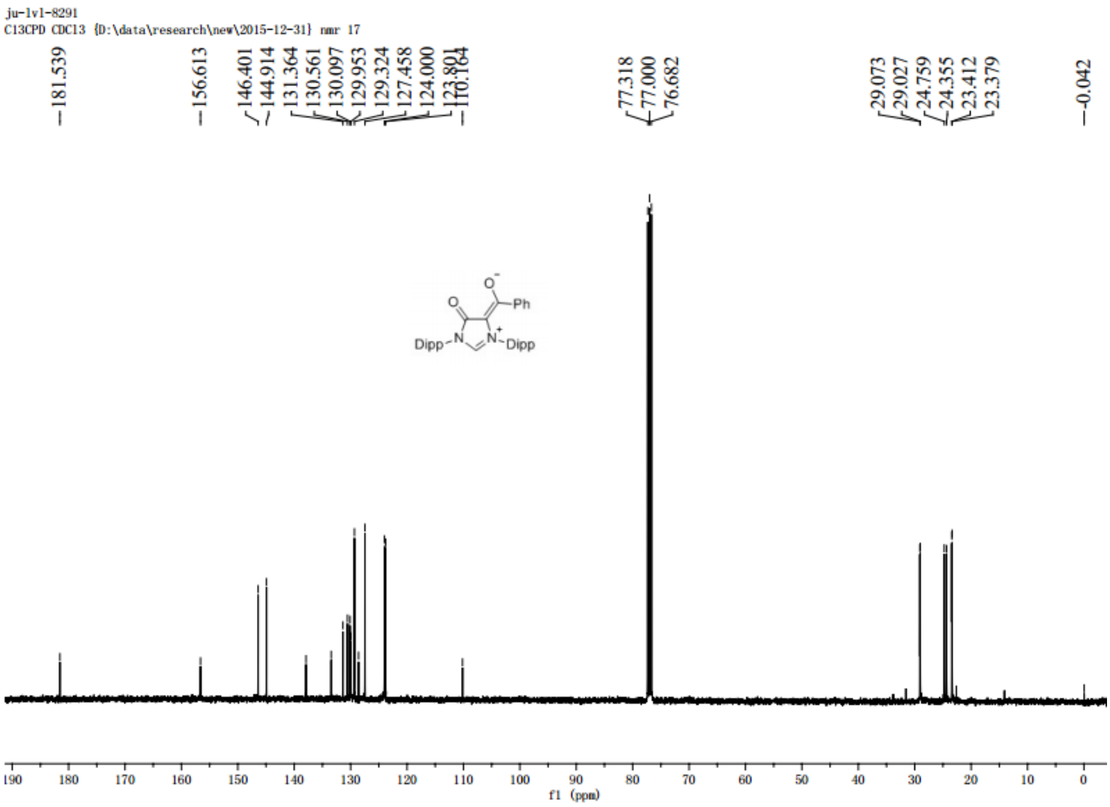

Supplementary Figure 11: ^{13}C-NMR Spectra of compound 6

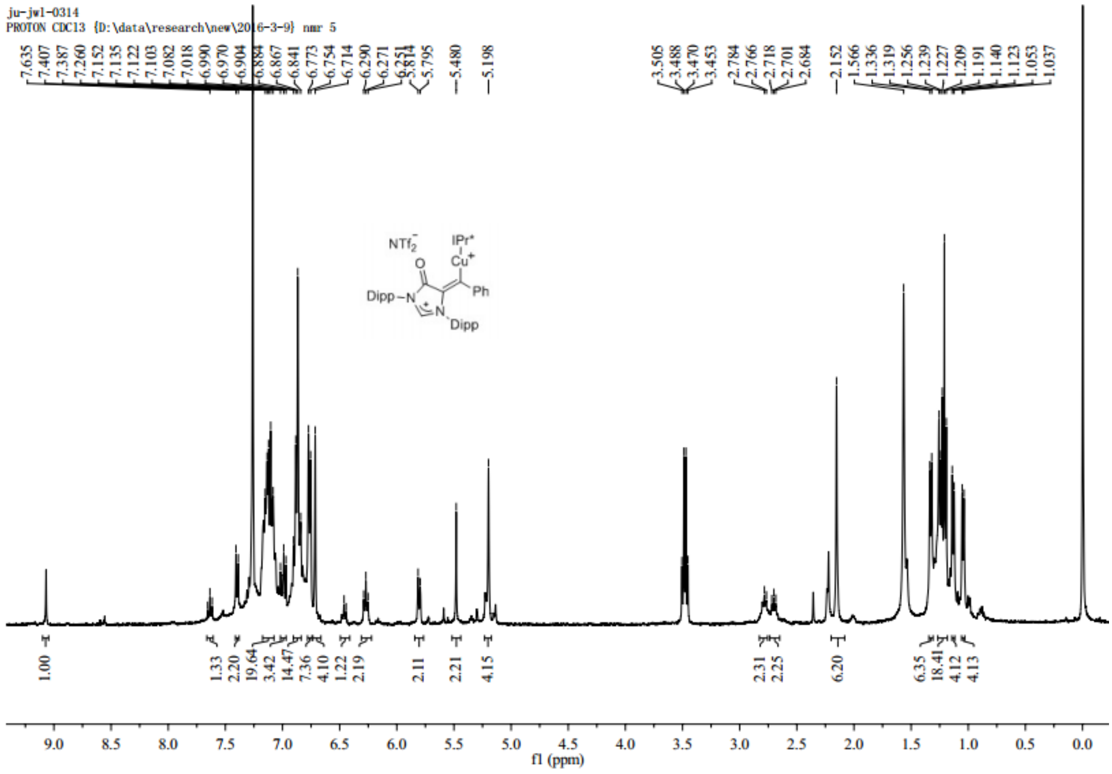

Supplementary Figure 12: ^{1}H-NMR Spectra of complex 7

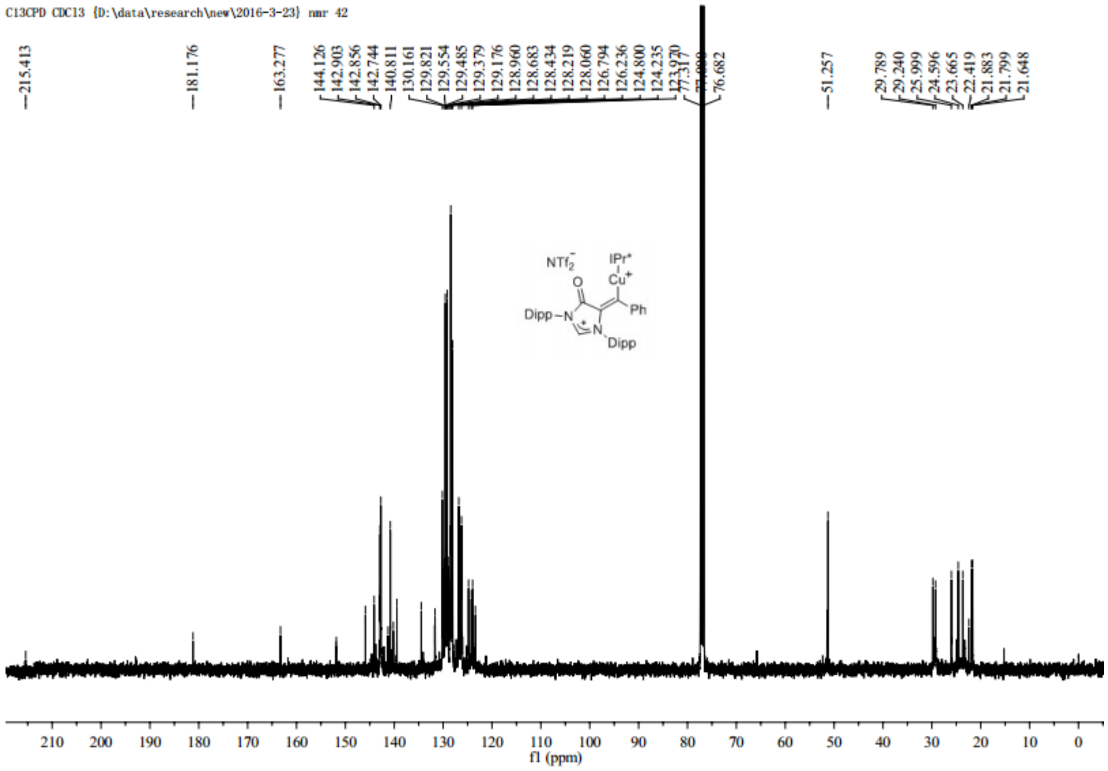

Supplementary Figure 13: ^{13}C-NMR Spectra of complex 7

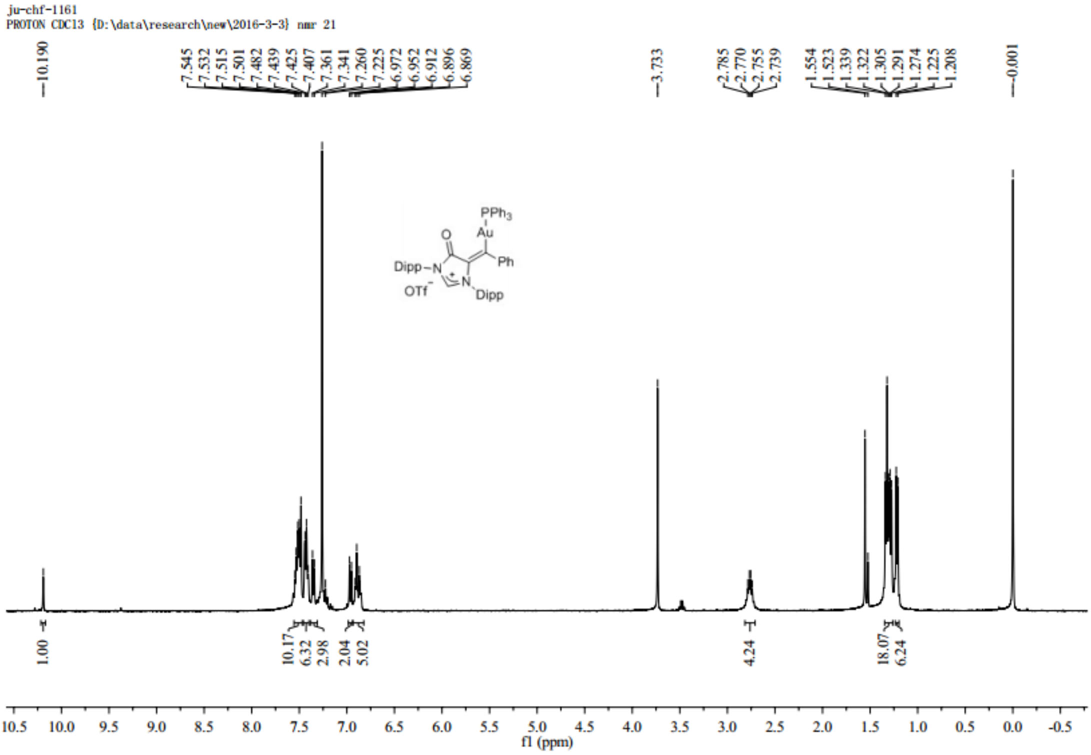

Supplementary Figure 14: ^{1}H-NMR Spectra of complex 8

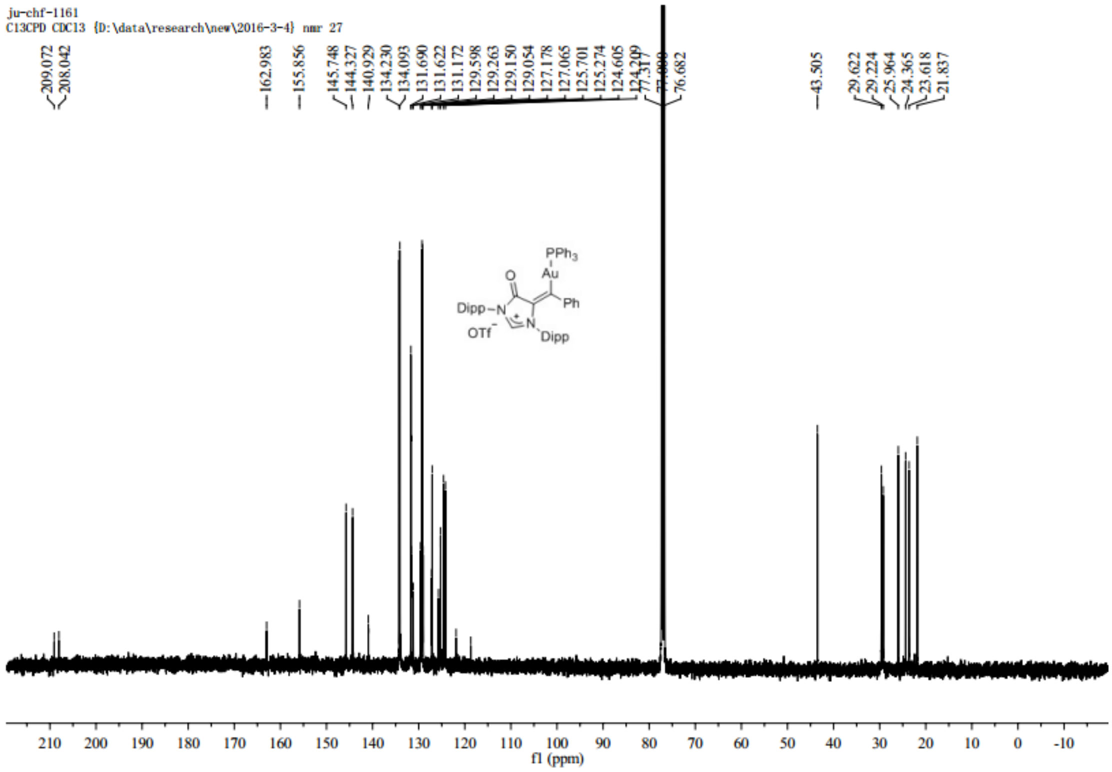

Supplementary Figure 15: ^{13}C-NMR Spectra of complex 8

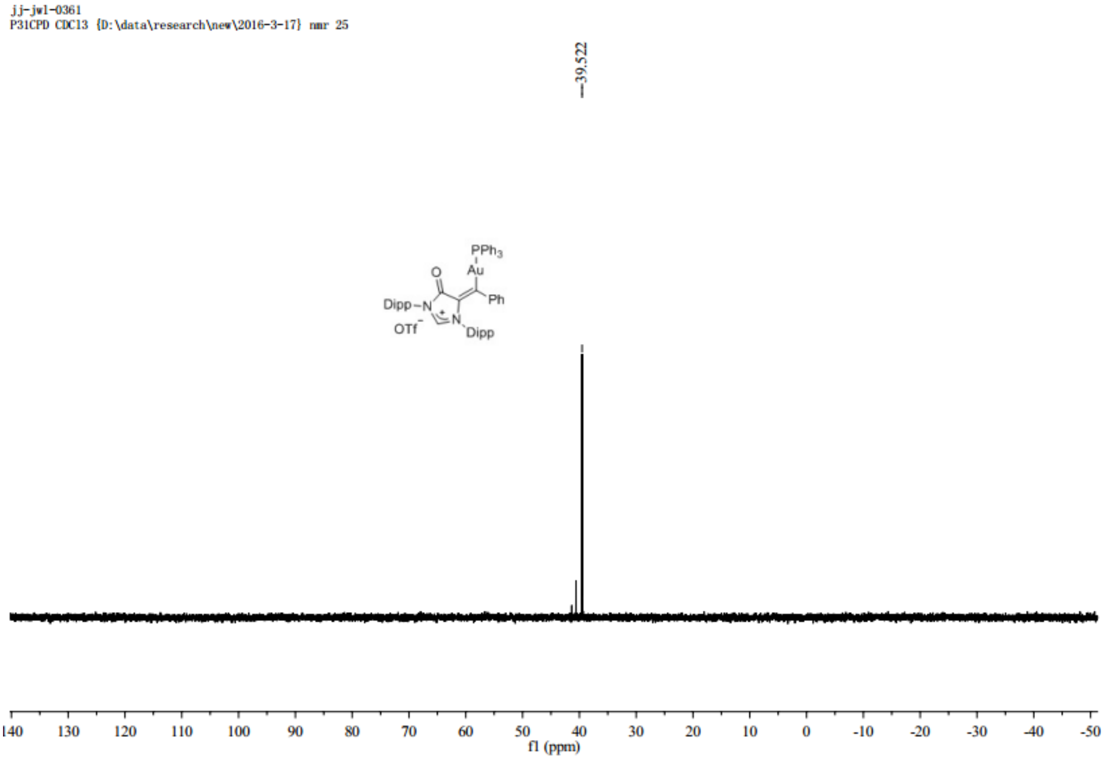

Supplementary Figure 16: ^{31}P-NMR Spectra of complex 8

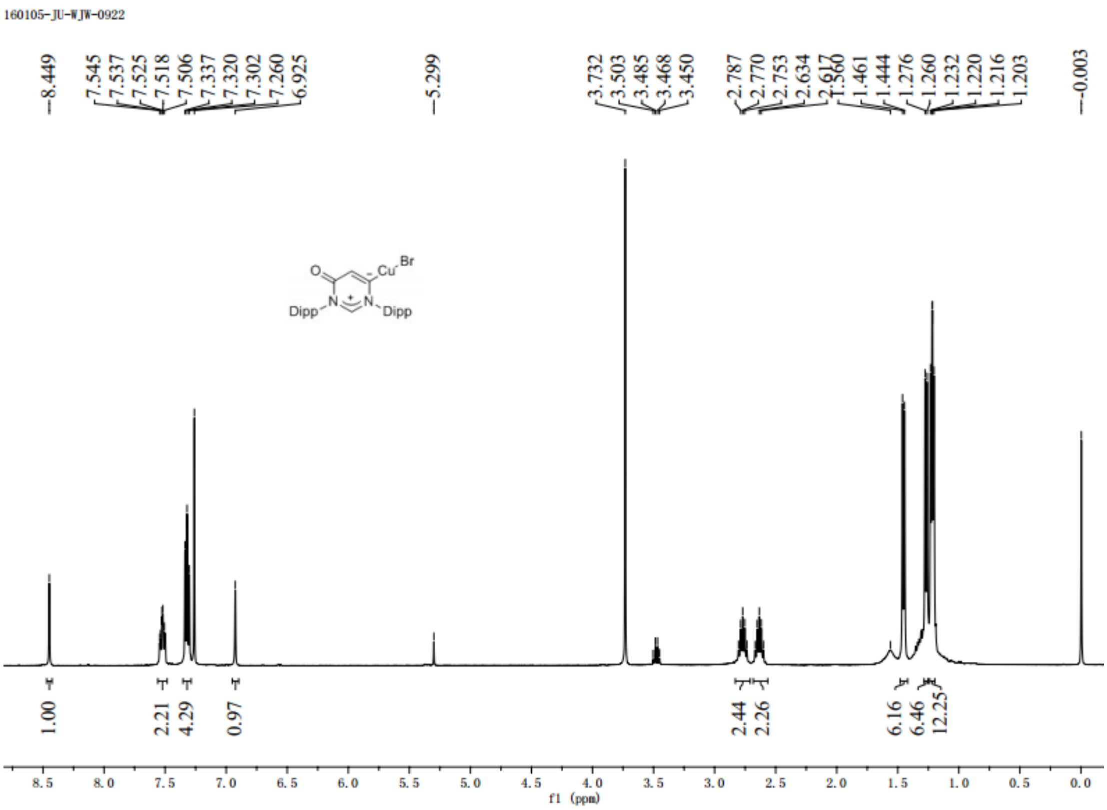

Supplementary Figure 17: ^{1}H-NMR Spectra of complex 18

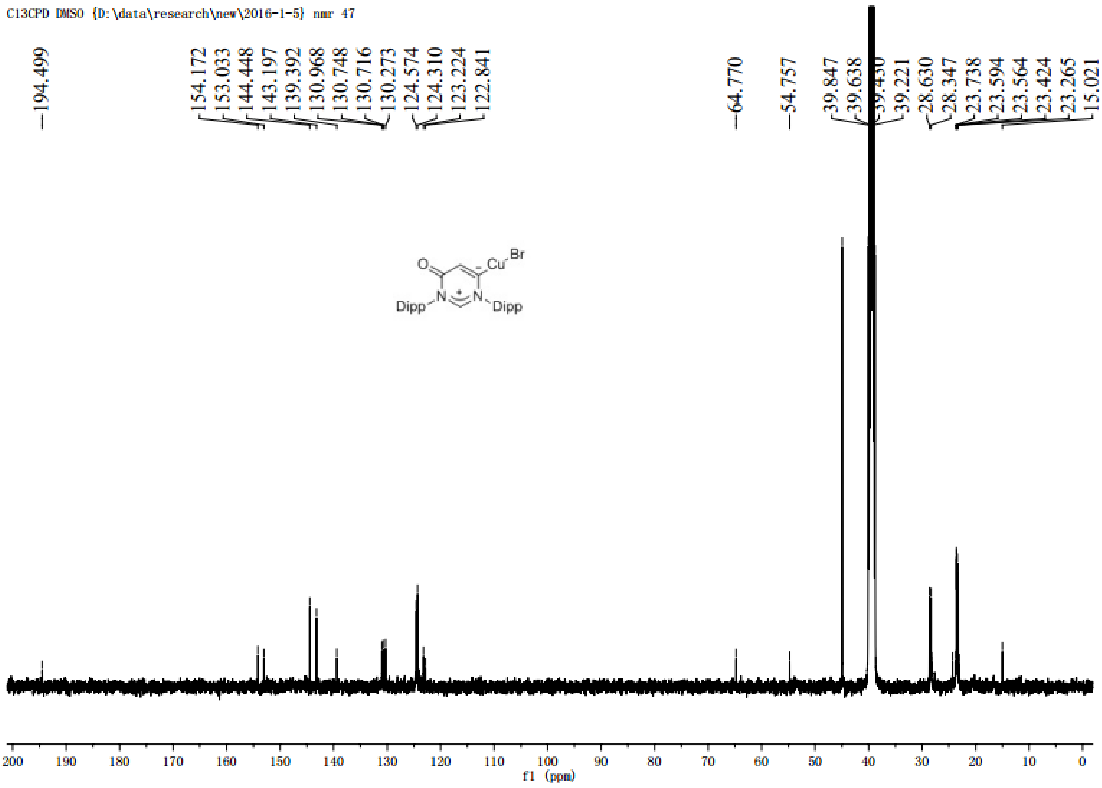

Supplementary Figure 18: ^{13}C-NMR Spectra of complex 18

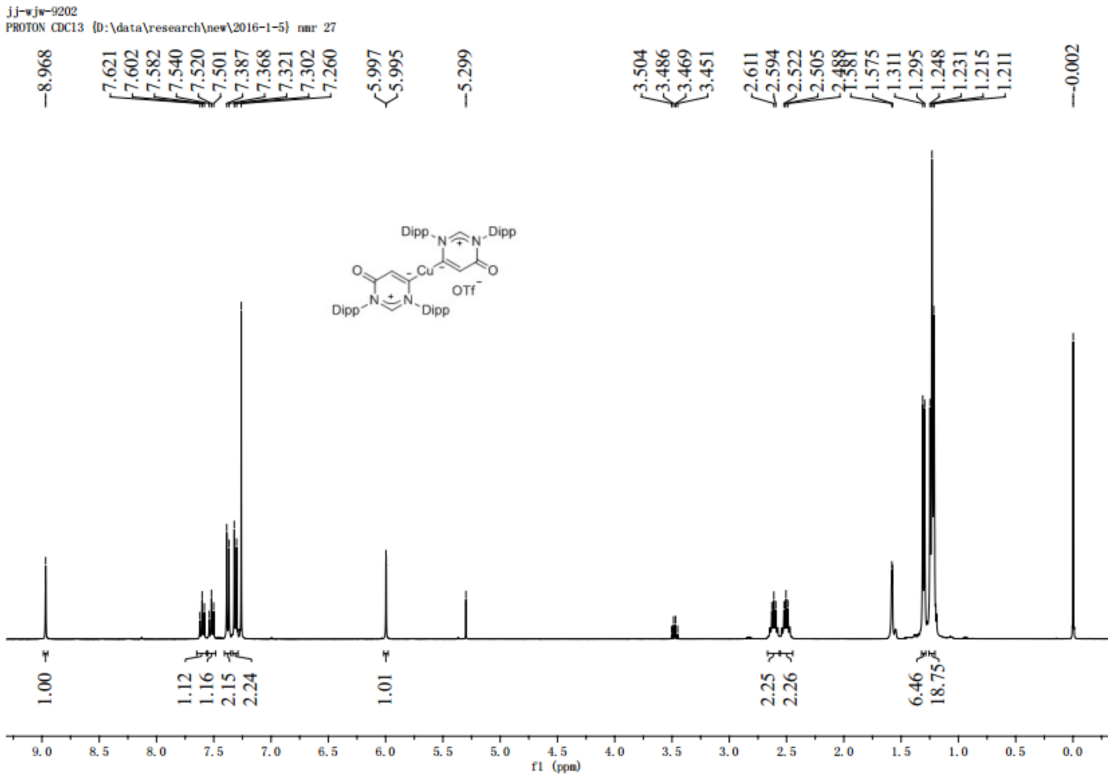

Supplementary Figure 19: ^{1}H-NMR Spectra of complex 19

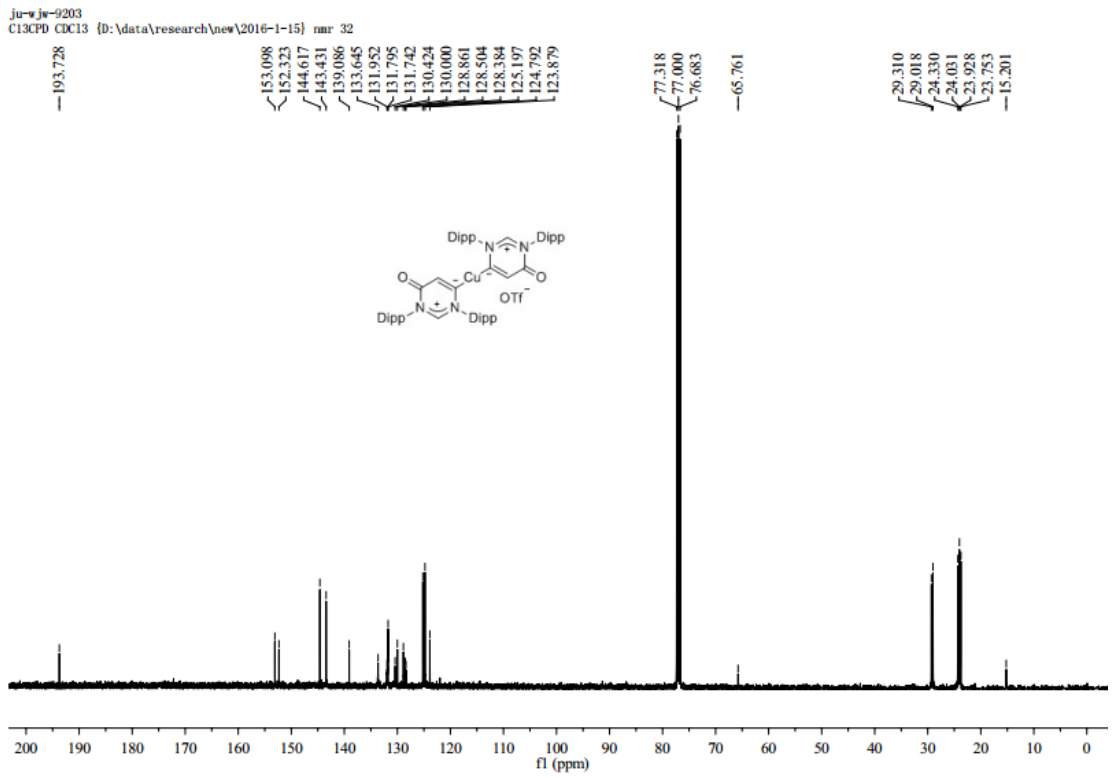

Supplementary Figure 20: ^{31}C-NMR Spectra of complex 19

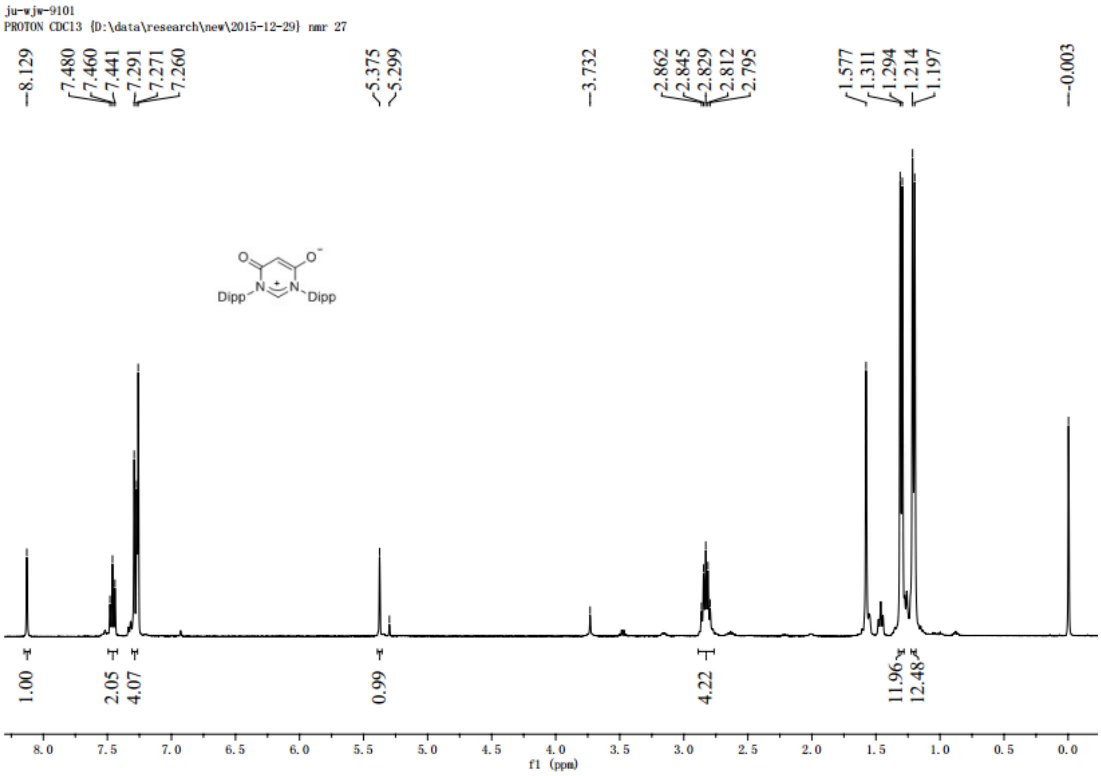

Supplementary Figure 21: ^{1}H-NMR Spectra of compound 20

Supplementary Methods

General Methods

Unless otherwise stated, all reactions and manipulations were performed using standard Schlenk techniques. All solvents were purified by distillation using standard methods. Commercially available reagents were used without further purification. NMR spectra were recorded by using a Bruker 400 MHz spectrometer. Chemical shifts are reported in ppm from tetramethylsilane with the solvent resonance as the internal standar (^{1}H NMR $CDCl_3$: 7.26 ppm; ^{13}C NMR $CDCl_3$: 77.0 ppm; ^{13}C NMR DMSO: 39.43 ppm). Mass spectra were recorded on the HP-5989 instrument by EI/ESI methods. X-ray diffraction analysis was performed by using a Bruker Smart-1000X-ray diffractometer. X-ray structure determination diffraction data of **6**, **7**, **8**, and **19** were collected on Bruker Smart APEX CCD diffract meter with graphite-monochromated Mo Kα radiation (λ = 0.71073 Å), and data of **4** and **5** were collected on Bruker APEX DUO diffract meter with graphite-monochromated Mo Kα radiation (λ = 0.71073 Å). These structures were solved by direct methods, using Fourier techniques, and refined on F2 by a full-matrix least-squares method. All the calculations were carried out with the SHELXTL program.[1]

Propiolic acid is commercially available and was used as received without further purification. **16**, **17** was synthesized by the procedures we previously reported.[2] IPrCuOTf[3] and IPr*$CuNTf_2$[4-6] were prepared according to the literature methods.

Preparation and characterization

Synthesis of formamidine 1

The mixture of *N,N'*-bis(2,6-diisopropylphenyl) formamidine (1.50 g, 4.12 mmol), phenylpropiolic acid (602mg, 4.12 mmol) and DCC (850 mg, 4.12 mmol) was stirred in the DCM (30 mL) at 0 °C. After stirred for 5 min, the crude product was purified by column chromatography using silica gel (v/v, PE/ EtOAc = 30:1) to afford **1** as a white solid (1.3g, 65%). ^{1}H NMR (400 MHz, $CDCl_3$) δ = 8.88 (s, 1H), 7.52 (t, J = 7.7 Hz, 1H), 7.36 (t, J = 7.8 Hz, 3H), 7.29-7.23 (m, 1H), 7.12-7.00 (m, 6H), 3.11-3.02 (m, 2H), 2.97-2.91 (m, 2H), 1.33-1.28 (m, 12H), 1.15 (d, J = 6.8 Hz, 12H); HRMS (ESI): m/z $[M+H]^+$ calcd. for

$C_{34}H_{41}N_2O^+$: 493.3219; found: 493.3203.

Synthesis of copper carbene 2

The mixture of **1** (100 mg, 0.20 mmol) and IPrCuOTf (120 mg, 0.20 mmol) was stirred in the DCE (2.5 ml) at 10 °C for 40 min. All volatiles were removed under vacuum, and the rude product was washed twice with diethyl ether to afford pure **2** as a purple solid (179 mg, 82%). ^{1}H NMR (400 MHz, $CDCl_3$) δ = 9.39 (s, 1H), 7.50 (t, J = 7.8 Hz, 1H), 7.40 (t, J = 7.8 Hz, 2H), 7.29 (s, 2H), 7.19 (d, J = 7.8 Hz, 4H), 7.13 (s, 3H), 6.86 (d, J = 7.8 Hz, 2H), 6.65 (t, J = 7.3 Hz, 1H), 6.54 (t, J = 7.3 Hz, 2H), 5.86 (d, J = 7.6 Hz, 2H), 2.56-2.46 (m, 8H), 1.22 (m, J = 6.8 Hz, 6H), 1.16 (d, J = 6.8 Hz, 12H), 1.13-1.01 (m, 30H); ^{13}C NMR (100 MHz, $CDCl_3$) δ = 211.38, 181.87, 162.48, 152.50, 145.66, 145.46, 144.18, 141.83, 134.63, 131.16, 129.97, 126.61, 126.07, 125.47, 124.45, 123.50, 123.27, 29.24, 28.88, 28.52, 25.84, 23.97, 23.48, 21.59; HRMS (MALDI): m/z $[M+H]^+$ calcd. for $C_{62}H_{77}CuF_3N_4O_4S^+$: 1093.4914; found: 1093.4914.

Synthesis of vinyl copper 3

The mixture of **2** (100 mg, 0.20 mmol) and IPrCuOTf (120 mg, 0.20 mmol) was stirred in the DCE (2.5 ml) at 60 °C for 30 min. All volatiles were removed under vacuum, and the rude product was washed twice with diethyl ether to afford pure **3** as a white solid (116 mg, 53%). ^{1}H NMR (400 MHz, $CDCl_3$) δ = 8.40 (s, 1H), 7.53-7.43 (m, 3H), 7.35 (t, J = 8.0 Hz, 1H), 7.28 (s, 3H), 7.23 (d, J = 8.0 Hz, 4H), 7.10-7.06 (m, 4H), 6.79-6.72 (m, 4H), 2.60-2.37 (m, 8H), 1.22 (d, J = 6.0 Hz, 6H), 1.17 (d, J = 6.8 Hz, 12H), 1.10-1.01 (m, 30H); ^{13}C NMR (100 MHz, DMSO) δ = 179.92, 161.63, 155.13, 154.25, 153.36, 150.45, 145.16, 144.84, 144.54, 143.94, 134.46, 131.75, 130.56, 130.66, 129.81, 128.82, 128.39, 127.38, 124.33, 123.69, 28.42, 28.06, 25.05, 23.97, 23.68, 23.01, 21.62; HRMS (MALDI): m/z $[M+H]^+$ calcd. for $C_{62}H_{77}CuF_3N_4O_4S^+$: 1093.4914; found: 1093.4928.

Synthesis of 5-membered cyclic formamidinium salt 4

From the IPrCuOTf-catalyzed cyclization of 4 in the presence of HOTf:

The mixture **1** (100 mg, 0.20 mmol) and trifluoromethanesulfonic acid (30 mg, 0.20

mmol) was stirred in the DCE (1.5 ml) at 90 °C, and then IPrCuOTf (6 mg, 0.01mmol) was added. After the solution was stirred for 45 min, all volatiles were removed under vacuum, and the rude product was washed twice with diethyl ether to afford pure **4** as a yellow solid (116 mg, 90%). ^{1}H NMR (400 MHz, $CDCl_3$) δ = 10.81 (s, 1H), 8.28 (d, J = 7.6 Hz, 2H), 7.73-7.67 (m, 2H), 7.61-7.53 (m, 3H), 7.47 (d, J = 7.9 Hz, 2H), 7.37 (d, J = 7.9 Hz, 2H), 7.05 (s, 1H), 2.69-2.60 (m, 4H), 1.38 (d, J = 6.9 Hz, 6H), 1.32 (d, J = 6.8 Hz, 6H), 1.28 (d, J = 6.8 Hz, 6H), 1.25 (d, J = 6.8 Hz, 6H); ^{13}C NMR (100 MHz, $CDCl_3$) δ = 158.92, 156.90, 146.03, 145.62, 142.07, 142.01, 136.18, 134.14, 133.26, 132.14, 129.78, 129.67, 126.00, 125.64, 125.22, 124.70, 29.90, 29.74, 24.25, 24.09, 23.72, 23.61; HRMS (ESI): m/z $[M\text{-}OTf]^+$ calcd. for $C_{34}H_{41}N_2O^+$: 493.3219; found: 493.3219.

From the reaction of 2 with HOTf:

The mixture of **2** (100 mg, 0.09 mmol) and trifluoromethanesulfonic acid (14 mg, 0.09 mmol) was stirred in the DCE (1.5 ml) at 25 °C for 10 min. All volatiles were removed under vacuum, and the rude product was washed twice with diethyl ether to afford pure **4** as a yellow solid (57 mg, 98%).

Synthesis of 6-membered cyclic formamidinium salt 5

The mixture of **3** (100 mg, 0.09 mmol) and trifluoromethanesulfonic acid (14 mg, 0.09 mmol) was stirred in the DCE (1.5 ml) at 10 °C for 10 min. All volatiles were removed under vacuum, and the rude product was washed twice with diethyl ether to afford pure **5** as a gray solid (55 mg, 95%). ^{1}H NMR (400 MHz, $CDCl_3$) δ = 10.48 (s, 1H), 7.60 (t, J = 7.8 Hz, 1H), 7.52 (t, J = 6.5 Hz, 1H), 7.50-7.46 (m, 1H), 7.41-7.32 (m, 4H), 7.24 (d, J = 7.8 Hz, 4H), 7.00 (s, 1H), 2.70-2.54 (m, 4H), 1.35 (d, J = 6.8 Hz, 6H), 1.28 (d, J = 6.8 Hz, 6H), 1.24 (d, J = 6.7 Hz, 6H), 1.05 (d, J = 6.7 Hz, 6H); ^{13}C NMR (100 MHz, $CDCl_3$) δ = 157.87, 156.53, 156.00, 144.53, 144.31, 132.93, 132.37, 132.15, 131.54, 129.24, 128.95, 128.83, 128.33, 125.49, 124.98, 116.30, 65.81, 29.60, 24.93, 24.67, 23.31, 22.58, 15.22; HRMS (ESI): m/z $[M\text{-}OTf]^+$ calcd. for $C_{34}H_{41}N_2O^+$: 493.3219; found: 493.3223.

Synthesis of compound 6

From the oxidation of vinylcopper species 2:

2 (30 mg, 0.03 mmol) was dissolved in the $CDCl_3$ (0.5 ml) at 25 °C. After standing for a week, a 34% NMR yield of **6** was observed by NMR spectroscopy.

From the cyclization of 1 catalyzed by CuBr·Me_2S:

The mixture of **1** (100 mg, 0.20 mmol) and CuBr·Me_2S (4 mg, 0.02 mmol) was stirred in the DCE (1.5 ml) at 25 °C. After stirring for 48 h, all volatiles were removed under vacuum, and the crude product was purified by column chromatography using silica gel (v/v, PE/ EtOAc = 4:1) to afford the pure **6** as a white solid (84 mg, 83%). ^1H NMR (400 MHz, $CDCl_3$) δ = 7.96 (d, J = 7.8 Hz, 2H), 7.46 (t, J = 7.8 Hz, 2H), 7.40 (d, J = 7.2 Hz, 1H), 7.38-7.26 (m, 7H), 2.95-2.80 (m, 4H), 1.35 (d, J = 6.8 Hz, 6H), 1.26-1.17 (m, 18H); ^{13}C NMR (100 MHz, $CDCl_3$) δ = 181.54, 156.61, 146.40, 144.91, 137.91, 133.45, 131.36, 130.56, 130.03, 129.32, 128.59, 127.46, 123.90, 110.16, 29.05, 24.76, 24.35, 23.40; HRMS (ESI): m/z $[M+H]^+$ calcd. for $C_{34}H_{41}N_2O_2^+$: 509.3168; found: 509.3162.

From the cyclization of 1 catalyzed by IPrCuOTf in the presence of H_2O_2:

The mixture of **1** (100 mg, 0.20 mmol), IPrCuOTf (12 mg, 0.02 mmol) and H_2O_2 (30%, 2.3 g, 20 mmol) was stirred in the DCE (1.5 ml) at 25 °C. After stirring for 12 h, all volatiles were removed under vacuum, and the crude product was purified by column chromatography using silica gel (v/v, PE/ EtOAc = 4:1) to afford the pure **6** as a white solid (58 mg, 57%).

From the cyclization of 1 catalyzed by IPr*$CuNTf_2$ in the presence of H_2O_2:

The mixture of **1** (100 mg, 0.20 mmol), IPr*$CuNTf_2$ (13 mg, 0.01 mmol) and H_2O_2 (30%, 2.3 g, 20 mmol) was stirred in the DCE (1.5 ml) at 10 °C. After stirring for 8 h, all volatiles were removed under vacuum, and the crude product was purified by column chromatography using silica gel (v/v, PE/ EtOAc = 4:1) to afford the pure **6** as a white solid (60 mg, 59%).

From the oxidation of copper carbene 7 in the presence of O_2:

7 (30 mg, 0.03 mmol) was dissolved in the $CDCl_3$ (0.5 ml) at 60 °C under oxygen. After

stirring for 5 h, a 36% NMR yield of **6** was observed by NMR spectroscopy.

From the cyclization of 1 catalyzed by PPh_3AuOTf in the presence of H_2O_2:

The mixture of **1** (100 mg, 0.20 mmol), PPh_3AuOTf (12 mg, 0.02 mmol) and H_2O_2 (30%, 2.3 g, 20 mmol) was stirred in the DCE (1.5 ml) at 25 °C. After stirring for 48 h, all volatiles were removed under vacuum, and the crude product was purified by column chromatography using silica gel (v/v, PE/ EtOAc = 4:1) to afford the pure **6** as a white solid (44 mg, 43%).

From the oxidation of gold carbene 8 in the presence of H_2O_2:

The mixture of **7** (110 mg, 0.10 mmol) and H_2O_2 (30%, 113 mg, 1.0 mmol) was stirred in the DCE (1.5 ml) at 25 °C. After stirring for 12 h, all volatiles were removed under vacuum, and the crude product was purified by column chromatography using silica gel (v/v, PE/ EtOAc = 4:1) to afford the pure **6** as a white solid (43 mg, 85%).

Synthesis of copper carbene 7

The mixture of **1** (100 mg, 0.20 mmol) and IPr^*CuNTf_2 (205 mg, 0.20 mmol) was stirred in the DCE (3 ml) at 10 °C for 30 min. All volatiles were removed under vacuum, and the rude product was washed twice with diethyl ether to afford pure **7** as a wine red solid (260 mg, 74%). ^{1}H NMR (400 MHz, $CDCl_3$) δ = 9.07 (s, 1H), 7.64 (t, J = 7.9 Hz, 1H), 7.40 (d, J = 7.9 Hz, 2H), 7.17-7.07 (m, 19H), 7.02-6.97 (m, 3H), 6.90-6.84 (m, 14H), 6.76 (d, J = 7.6 Hz, 7H), 6.71 (s, 4H), 6.45 (t, J = 6.8 Hz, 1H), 6.27 (t, J = 7.6 Hz, 2H), 5.80 (d, J = 7.6 Hz, 2H), 5.48 (s, 2H), 5.20 (s, 4H), 2.83-2.76 (m, 2H), 2.74-2.65 (m, 2H), 2.15 (s, 6H), 1.33 (d, J = 6.8 Hz, 6H), 1.27-1.18 (m, 12H), 1.13 (d, J = 6.8 Hz, 3H), 1.05 (d, J = 6.8 Hz, 3H); ^{13}C NMR (100 MHz, $CDCl_3$) δ = 215.41, 181.18, 163.28, 151.88, 145.91, 144.13, 143.18, 142.38, 140.81, 140.19, 139.46, 134.48, 131.66, 129.99, 129.55, 129.43, 129.38, 129.18, 128.68, 128.43, 128.06, 126.79, 126.55, 126.24, 124.80, 124.57, 124.41, 124.24, 123.69, 29.79, 29.24, 26.00, 24.60, 23.66, 22.42, 21.99, 21.36; HRMS (MALDI): m/z $[M-NTf_2]^+$ calcd. for $C_{103}H_{96}CuN_4O^+$: 1467.6880; found: 1467.6895.

Synthesis of gold carbene 8

The mixture of PPh_3AuCl (99 mg, 0.20 mmol) and silver triflate (51 mg, 0.20 mmol) was stirred in the DCE (1.5 mL) at 25 °C for 15 minutes, then the solid components were filtered off and the filtrate was added to the solution of **1** (100 mg, 0.20 mmol) in the DCE (1 mL). After stirring for 1 h at 25 °C, all volatiles were removed under vacuum . The rude product was washed twice with diethyl ether to afford pure **8** as a yellow solid (132 mg, 60%). ^{1}H NMR (400 MHz, $CDCl_3$) δ = 10.19 (s, 1H), 7.56-7.47 (m, 10H), 7.46-7.39 (m, 6H), 7.35 (d, J = 7.9 Hz, 3H), 6.96 (d, J = 7.9 Hz, 2H), 6.93-6.83 (m, 5H), 2.84-2.67 (m, 4H), 1.39-1.26 (m, 18H), 1.22 (d, J = 6.8 Hz, 6H); ^{13}C NMR (100 MHz, $CDCl_3$) δ = 208.56 (d, $^2J_{C\text{-}P}$ = 103.0 Hz), 162.98, 155.86, 145.75, 144.33, 140.93, 134.16, 131.66, 131.17, 129.60, 129.16, 127.12, 125.70, 125.27, 124.61, 124.21, 121.89, 29.62, 29.22, 25.96, 24.36, 23.62, 21.84; ^{31}P NMR (162 MHz, $CDCl_3$) δ = 39.52; HRMS (MALDI): m/z $[M\text{-}OTf]^+$ calcd. for $C_{52}H_{55}AuN_2OP^+$: 951.3718; found: 951.3699.

The oxidation reaction of complex 16 in the presence of H_2O_2:

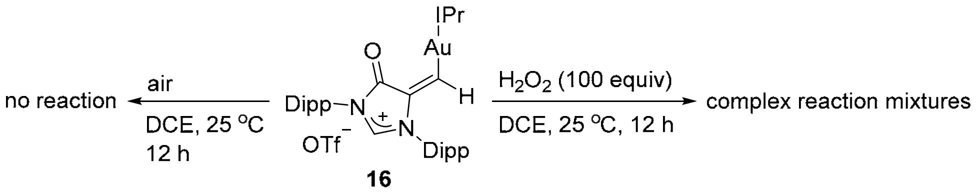

Supplementary Figure 22. The reactivity of vinyl gold complex 16 towards oxidation.

The mixture of **16** (50 mg, 0.04 mmol) and H_2O_2 (30%, 453 mg, 4.0 mmol) was stirred in the DCE (1.5 ml) at 25 °C. After stirring for 12 h, all volatiles were removed under vacuum, and the reaction mixture was determined by ^{1}H NMR analysis.

Synthesis of 6-membered copper complex 18

The mixture of **17** (100 mg, 0.24 mmol) and ethyldiisopropylamine (31 mg, 0.24 mmol) was stirred in the DCE (2 ml) at 25 °C, and then $CuBr{\cdot}Me_2S$ (49 mg, 0.24 mmol) was added. After stirring for 20 min, all volatiles were removed under vacuum, and the resultant solid was solvents in DCM and filtered through a pad of Celite. After washing the solid several times with DCM, all volatiles were removed under vacuum, and the rude product was washed

twice with diethyl ether to afford pure **18** as a yellow solid (87 mg, 65%). ^{1}H NMR (400 MHz, $CDCl_3$) δ = 8.45 (s, 1H), 7.56-7.48 (m, 2H), 7.35-7.29 (m, 4H), 6.93 (s, 1H), 2.83-2.71 (m, 2H), 2.68-2.56 (m, 2H), 1.45 (d, J = 6.7 Hz, 6H), 1.27 (d, J = 6.7 Hz, 6H), 1.24-1.19 (m, 12H); ^{13}C NMR (100 MHz, DMSO) δ = 194.50, 154.17, 153.60, 143.82, 139.39, 130.81, 124.57, 124.31, 28.37, 24.20, 23.08; HRMS (MALDI): m/z $[M-Br]^+$ calcd. for $C_{28}H_{36}CuN_2O^+$: 479.2124; found: 479.2117.

Synthesis of copper complex 19

The mixture of triphenylphosphine (47 mg, 0.18 mmol) and silver triflate (46 mg, 0.18 mmol) was stirred in the DCE (2 ml) at 25 °C for 15 min, and then **18** (100 mg, 0.18 mmol) was added. After stirring for 2 h, all volatiles were removed under vacuum, and the resultant solid was solvents in DCM and filtered through a pad of Celite. After washing the solid several times with DCM, all volatiles were removed under vacuum, the yellow solid was washed twice with diethyl ether to afford a rude product, which was purified by column chromatography using silica gel (v/v, DCM/ EtOH = 400:1) to afford pure **19** as a white solid (60 mg, 64%). ^{1}H NMR (400 MHz, $CDCl_3$) δ = 8.97 (s, 1H), 7.60 (t, J = 7.8 Hz, 1H), 7.52 (t, J = 7.8 Hz, 1H), 7.38 (d, J = 7.8 Hz, 2H), 7.31 (d, J = 7.8 Hz, 2H), 6.00 (d, J = 0.7 Hz, 1H), 2.67-2.56 (m, 2H), 2.55-2.45 (m, 2H), 1.30 (d, J = 6.8 Hz, 6H), 1.26-1.20 (m, 18H); ^{13}C NMR (100 MHz, $CDCl_3$) δ = 193.73, 153.10, 152.32, 144.62, 143.43, 139.09, 133.64, 132.14, 131.50, 130.21, 129.96, 129.81, 128.86, 128.44, 125.20, 124.79, 123.88, 29.31, 29.02, 24.33, 24.18, 23.61; HRMS (ESI): m/z $[M-OTf]^+$ calcd. for $C_{56}H_{72}CuN_4O_2^+$: 895.4951; found: 895.4926.

Synthesis of compound 20

18 (100 mg, 0.18 mmol) was stirred in the DCE (1 ml) at 25 °C under air. After stirring for 24 h, all volatiles were removed under vacuum, the resultant solid was solvents in DCM, and filtered through a pad of Celite. After washing the solid several times with DCM, all volatiles were removed under vacuum, the rude product was washed twice with pentane to afford pure **20** as a brown solid (63 mg, 81%). The NMR analysis data of **18** are in full agreement with those reported in the literature.[7]

Computational Details

All the density functional theory (DFT) calculations were performed using Gaussian 09 suite of program[8]. The TPSS functional[9] with Grimme's D3-BJ[10,11] correction for van der Waals interaction was utilized in combination with the triple-ζ basis set def2-TZVPP[12,13], which has recently been shown to yield good structural parameters for Au complexes[14,15]. Solvent corrections were considered based on the integral equation formalism version of polarizable continuum model (IEF-PCM)[16-18] for CH_2Cl_2 (ε = 8.93) during the geometries optimization of the gold-carbene complexes. The bond orders of complexes were analyzed based on Mayor method[19] utilizing Multiwfn[20]. Intrinsic bond orbital (IBO)[21] analyses were made using IboView[22].

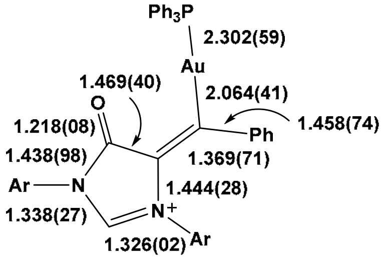

Supplementary Figure 23. Optimized Geometry Structure 8 (CH_2Cl_2)

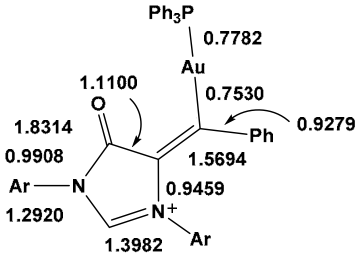

Supplementary Figure 24. Mayer Bonding Order Analysis of 8

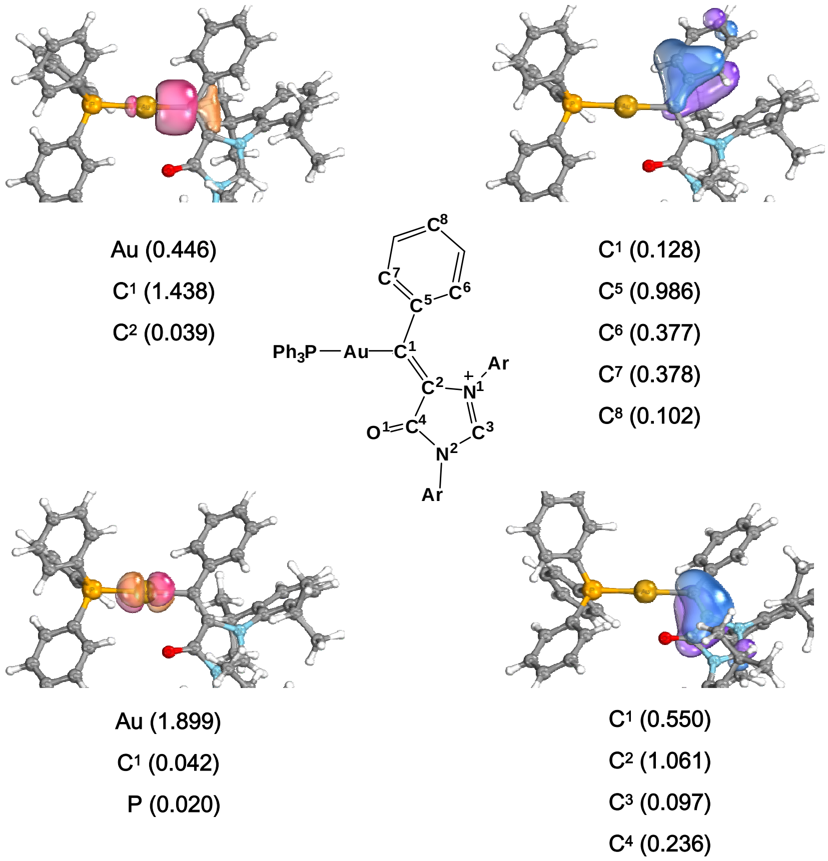

Supplementary Figure 25. C^1-stabilizing IBOs of gold complex 8. Numbers in parentheses indicate the fraction of electrons of the doubly occupied orbital assigned to the individual atoms.

As depicted in Figure S3, we identified a strong π-stabilization in **8**, which is mainly achieved through the π system of the imidazolium-4-olate ring attached to C^1 (Figure S3, bottom right). Additionally, small contribution from the phenyl ring attached to C^1 was also identified. The phenyl ring is polarized towards C^1 (Figure S3, top right), forming the delocalized π bonding with C^1. In addition, the IBO of coordinative bond between the lone pair of carbene C^1 and the gold atom was identified (Figure S3, top left) since this IBO is mainly located at C^1. The IBO representing the filled d orbital at gold, aligned for π backbonding was also identified but it is largely located at gold atom up to 96.8% (Figure S3, bottom left), suggesting little contribution to stabilize carbenic C^1.

X-Ray Crystallography.

Key details of the crystal and structure refinement data are summarized in Table S4-S5. Further crystallographic details may be found in the respective CIF files, which were deposited at the Cambridge Crystallographic Data Centre, Cambridge. CCDC 1418063 (**4**), CCDC 1418064 (**5**), CCDC 1449714 (**6**), CCDC 1470533 (**7**), CCDC 1470532 (**8**), and CCDC 1449046 (**19**) contain the supplementary crystallographic data for this paper. These data can be obtained free of charge from The Cambridge Crystallographic Data Centre via www.ccdc.cam.ac.uk/data_request/cif.

In the structure of compound **4**, the solvent accessible voids occupy volumes of 438 Å^3, and a total of 44 electrons were found in each cell. The void spaces are filled with unidentifiable electron densities (tentatively assumed as four water molecules based on the ^{1}H NMR spectra), and their contributions to the scattering factors were removed by SQUEEZE.[23]

The structure of compound **5** is twinned about [0 0 1] lattice direction and the twin matrix was suggested by ROTAX program while the HKLF 5 type file was prepared using MAKE HKLF5 in WinGX suite.[24] The ratio of two twinned domains was fined to 0.21/0.79.

In the structure of complex **7**, the NTf_2 anion displayed positional disorder with the relative ratio of 0.65/0.35 refined for the two components. The site occupancy for the CH_2Cl_2 solvate was fixed at 0.5 to obtain reasonable thermal factors. A small amount of spatially delocalized electron density in the lattice was found but acceptable refinement results could not be obtained for this electron density. The solvent contribution was then modeled using SQUEEZE in the Platon program suite. The solvent accessible voids occupy volumes of 536 Å^3, and a total of 94 electrons were found in each cell. The void spaces are filled with unidentifiable electron densities (tentatively assumed as one CH_2Cl_2 molecule based on the ^{1}H NMR spectra), and their contributions to the scattering factors were removed by SQUEEZE.[24]

In the structure of complex **8**, the solvent accessible voids occupy volumes of 497 Å^3, and a total of 88 electrons were found in each cell. The void spaces are filled with unidentifiable electron densities (tentatively assumed as one CH_2Cl_2 molecule based on the ^{1}H NMR spectra), and their contributions to the scattering factors were removed by SQUEEZE.[24]

In the structure of complex **19**, the solvent accessible voids occupy volumes of 379 Å^3, and a total of 83 electrons were found in each cell. The void spaces are filled with

unidentifiable electron densities (tentatively assumed as two CH_2Cl_2 molecules based on the ^{1}H NMR spectra), and their contributions to the scattering factors were removed by SQUEEZE.[24]

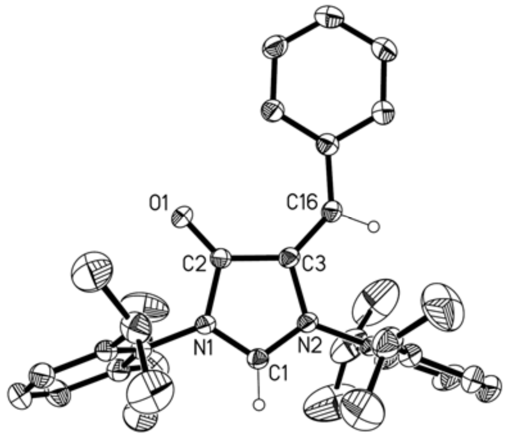

Supplementary Figure 26. Molecular structure of 4 with 20% probability. The counterion (OTf^-) and H atoms in aryl rings have been omitted for clarity.

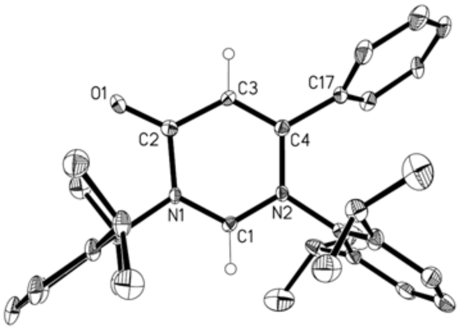

Supplementary Figure 27. Molecular structure of 5 with 20% probability. The counterion (OTf^-) and H atoms in aryl rings have been omitted for clarity.

Supplementary Table 1. Crystal Data, Data Collection, and Structure Refinement for **4, 5** and **6**.

	**4**	**5**	**6**
Identification code	mo_50520b	mo_50424a	a60121a
Formula	$C_{35}H_{41}F_3N_2O_4S$	$C_{35}H_{41}F_3N_2O_4S$	$C_{17}H_{20}NO$
Formula weight	642.76	642.76	254.34
T, K	203(2)	173(2)	298(2)
crystal system	Monoclinic	Monoclinic	Triclinic
space group	P 21/c	P 21/n	P -1
a, Å	9.0556(16)	10.959(3)	11.537(18)
b, Å	18.043(3)	21.509(6)	11.605(18)
c, Å	23.391(4)	14.137(4)	11.950(19)
α, deg	90	90	74.12(2)
β, deg	91.684(3)	96.106(5)	87.37(2)
γ, deg	90	90	82.09(2)
Volume, $Å^3$	3820.3(12)	3313.7(16)	1524(4)
Z	4	4	4
D_{calc}, Mg / m^3	1.118	1.288	1.108
absorption coefficient, mm^{-1}	0.134	0.155	0.068
F(000)	1360	1360	548
crystal size, mm	0.400 x 0.300 x 0.200	0.420 x 0.280 x 0.220	0.400 x 0.250 x 0.150
2θ range, deg	2.076 to 26.998	1.731 to 25.999	1.772 to 25.007
reflections collected /unique	26664/8310 [R(int) = 0.0434]	21898/6874 [R(int) = 0.0530]	6407/5260 [R(int) = 0.0599]
data / restraints/ parameters	8310 / 360 / 551	20948 / 0 / 415	5260 / 0 / 355
goodness of fit on F^2	1.035	1.043	0.833
final R indices [$I > 2\sigma(I)$][a]	R1 = 0.0800, wR2 = 0.2465	R1 = 0.0942, wR2 = 0.2472	R1 = 0.0617, wR2 = 0.1495
R indices (all data)	R1 = 0.1345, wR2 = 0.2866	R1 = 0.1410, wR2 = 0.2878	R1 = 0.1218, wR2 = 0.1678
lgst diff peak and hole, e/$Å^3$	0.446 and -0.307	2.028 and -0.870	0.181 and -0.197

Supplementary Table 2. Crystal Data, Data Collection, and Structure Refinement for **7, 8** and **19**

	**8**	**19**	**7**
Identification code	a60309c	a60108a	a60323a
Formula	$C_{54}H_{57}AuCl_2F_3N_2O_4PS$	$C_{57}H_{72}CuF_3N_4O_5$ S	$C_{211}H_{194}Cl_2Cu_2F_{12}N_{10}O_{10}S_4$
Formula weight	1100.98	1045.78	3583.97
T, K	293(2)	298(2)	293(2) K
crystal system	Triclinic	Triclinic	Triclinic
space group	P-1	P -1	P-1
a, Å	8.865(4)	10.725(10)	13.813(5)
b, Å	16.621(8)	11.047(10)	17.708(6)
c, Å	19.292(10)	16.469(15)	21.514(7)
α, deg	92.140(6)	98.908(14)	78.667(5)
β, deg	92.137(6)	108.973(14)	80.265(5)
γ, deg	97.840(6)	100.921(14)	78.271(5)
Volume, $Å^3$	2811(2)	1762(3)	5006(3)
Z	2	1	1
D_{calc}, Mg / m^3	1.401	0.986	1.189
absorption coefficient, mm^{-1}	2.830	0.386	0.350
F(000)	1196	554	1874
crystal size, mm	0.400 x 0.200 x 0.200	0.180 x 0.060 x 0.040	0.400 x 0.300 x 0.200
2θ range, deg	1.238 to 25.249	1.345 to 25.010	1.519 to 25.250
reflections collected /unique	13773/9825 [R(int) = 0.0277]	7437/6092 [R(int) = 0.0637]	21535/17670 [R(int) = 0.0496]
data / restraints/ parameters	9825 / 36 / 594	6092 / 39 / 348	17670 / 52 / 1104
goodness of fit on F^2	0.989	1.104	1.046
final R indices [$I > 2\sigma(I)$]^a	R1 = 0.0621, wR2 = 0.1816	R1 = 0.1388, wR2 = 0.3887	R1 = 0.0943, wR2 = 0.2227
R indices (all data)	R1 = 0.0828, wR2 = 0.2123	R1 = 0.2129, wR2 = 0.4112	R1 = 0.1730, wR2 = 0.2393
lgst diff peak and hole, e/$Å^3$	2.384 and -2.108	1.743 and -0.601	1.457 and -0.825

Supplementary References:

1. Sheldrick, G. M. SHELXL-97 (Univ. Göttingen, 1997).
2. Lv, S. *et al.* Silver-catalyzed amidiniumation of alkynes: isolation of a silver intermediate, synthesis of enamine amido carbene precursors, and an unprecedented umpolung of propiolamide. *Angew. Chem., Int. Ed.* **54,** 14941-14946 (2015).
3. Cheng, L.-J. & Cordier, C. J. Catalytic nucleophilic fluorination of secondary and tertiary propargylic electrophiles with a copper-N-heterocyclic carbene complex. *Angew. Chem., Int. Ed.* **54,** 13734-13738 (2015).
4. Berthon-Gelloz, G. *et al.* IPr* an easily accessible highly hindered N-heterocyclic carbene. *Dalton Trans.* **39,** 1444-1446 (2010).
5. Gómez-Suárez, A. *et al.* Influence of a very bulky N-heterocyclic carbene in gold-mediated catalysis. *Organometallics* **30,** 5463-5470 (2011).
6. Hussong, M. W., Hoffmeister, W. T., Rominger, F. & Straub, B. F. Copper and silver carbene complexes without heteroatom stabilization: structure, spectroscopy, and relativistic effects. *Angew. Chem., Int. Ed.* **54,** 10331-10335 (2015).
7. Mushinski, R. M., Squires, B. M., Sincerbox, K. A. & Hudnall, T. W. Amino-acrylamido carbenes: modulating carbene reactivity via decoration with an *α,β*-unsaturated carbonyl moiety. *Organometallics* **31,** 4862-4870 (2012).
8. Frisch, M. J. *et al.* Gaussian 09 (Revision D.01), Gaussian, Inc., Wallingford, CT, 2009.
9. Tao, J., Perdew, J. P., Staroverov, V. N. & Scuseria, G. E. Climbing the density functional ladder: nonempirical meta-generalized gradient approximation designed for molecules and solids. *Phys, Rev. Lett.* **91,** 146401-146404 (2003).
10. Weigend, F. & Ahlrichs, R. Balanced basis sets of split valence, triple zeta valence and quadruple zeta valence quality for H to Rn: design and assessment of accuracy. *Phys. Chem. Chem. Phys.* **7,** 3297-3305 (2005).
11. Weigend, F. Accurate coulomb-fitting basis sets for H to Rn. *Phys. Chem. Chem. Phys.* **8,** 1057-1065 (2006).
12. Grimme, S., Antony, J., Ehrlich, S. & Krieg, H. A consistent and accurate ab initio parametrization of density functional dispersion correction (DFT-D) for the 94 elements H-Pu. *J. Chem. Phys.* **132,** 154104-154119 (2010).

13. Grimme, S., Ehrlich, S. & Goerigk, L. Effect of the damping function in dispersion corrected density functional theory. *J. Comput. Chem.* **32,** 1456-1465 (2011).

14. Nava, P., Hagebaum-Reignier, D. & Humbel, S. Bonding of gold with unsaturated species. *ChemPhysChem* **13,** 2090-2096 (2012).

15. Comprido, L. N. S., Klein, J. E. M. N., Knizia, G., Kästner, J. & Hashmi, A. S. K. The stabilizing effects in gold carbene complexes. *Angew. Chem. Int. Ed.* **54,** 10336-10340 (2015).

16. Miertuš, S., Scrocco, E. & Tomasi, J. Electrostatic interaction of a solute with a continuum. A direct utilizaion of AB initio molecular potentials for the prevision of solvent effects. *Chem. Phys.* **55,** 117-129 (1981).

17. Barone, V. & Cossi, M. Quantum calculation of molecular energies and energy gradients in solution by a conductor solvent model. *J. Phys. Chem. A* **102,** 1995-2001 (1998).

18. Cossi, M. & Barone, V. Separation between fast and slow polarizations in continuum solvation models. *J. Phys. Chem. A* **104,** 10614-10622 (2000).

19. Mayer, I. Improved definition of bond orders for correlated wave functions. *Chem. Phys. Lett.* **544**, 83-86 (2012).

20. Lu, T. & Chen, F. Multiwfn: a multifunctional wavefunction analyzer. *J. Comp. Chem*. **33,** 580-592 (2012).

21. Knizia, G. Intrinsic atomic orbitals: an unbiased bridge between quantum theory and chemical concepts. *J. Chem. Theory Comput.* **9,** 4834-4843 (2013).

22. Knizia, G. IboView. http://www.iboview.org.

23. Spek, A. L. Single-crystal structure validation with the program PLATON. *J. Appl. Cryst.* **36,** 7-13 (2003).

24. Farrugia, L. J. *J. Appl. Cryst*. WinGX and ORTEP for windows: an update. **45,** 849-854 (2012).
